# Phylogenetic Dependency Networks: Inferring Patterns of CTL Escape and Codon Covariation in HIV-1 Gag

**DOI:** 10.1371/journal.pcbi.1000225

**Published:** 2008-11-21

**Authors:** Jonathan M. Carlson, Zabrina L. Brumme, Christine M. Rousseau, Chanson J. Brumme, Philippa Matthews, Carl Kadie, James I. Mullins, Bruce D. Walker, P. Richard Harrigan, Philip J. R. Goulder, David Heckerman

**Affiliations:** 1 eScience Group, Microsoft Research, Redmond, Washington, United States of America; 2 Department of Computer Science and Engineering, University of Washington, Seattle, Washington, United States of America; 3 Partners AIDS Research Center, Massachusetts General Hospital, Harvard Medical School, Boston, Massachusetts, United States of America; 4 Department of Microbiology, University of Washington, Seattle, Washington, United States of America; 5 Department of Paediatrics, Nuffield Department of Medicine, University of Oxford, Oxford, United Kingdom; 6 Department of Medicine, University of Washington, Seattle, Washington, United States of America; 7 Howard Hughes Medical Institute, Chevy Chase, Maryland, United States of America; 8 B.C. Centre for Excellence in HIV/AIDS, Vancouver, British Columbia, Canada; 9 Department of Medicine, University of British Columbia, Vancouver, British Columbia, Canada; 10 HIV Pathogenesis Programme, The Doris Duke Medical Research Institute, University of KwaZulu-Natal, Durban, South Africa; Utrecht University, The Netherlands

## Abstract

HIV avoids elimination by cytotoxic T-lymphocytes (CTLs) through the evolution of escape mutations. Although there is mounting evidence that these escape pathways are broadly consistent among individuals with similar human leukocyte antigen (HLA) class I alleles, previous population-based studies have been limited by the inability to simultaneously account for HIV codon covariation, linkage disequilibrium among HLA alleles, and the confounding effects of HIV phylogeny when attempting to identify HLA-associated viral evolution. We have developed a statistical model of evolution, called a phylogenetic dependency network, that accounts for these three sources of confounding and identifies the primary sources of selection pressure acting on each HIV codon. Using synthetic data, we demonstrate the utility of this approach for identifying sites of HLA-mediated selection pressure and codon evolution as well as the deleterious effects of failing to account for all three sources of confounding. We then apply our approach to a large, clinically-derived dataset of Gag p17 and p24 sequences from a multicenter cohort of 1144 HIV-infected individuals from British Columbia, Canada (predominantly HIV-1 clade B) and Durban, South Africa (predominantly HIV-1 clade C). The resulting phylogenetic dependency network is dense, containing 149 associations between HLA alleles and HIV codons and 1386 associations among HIV codons. These associations include the complete reconstruction of several recently defined escape and compensatory mutation pathways and agree with emerging data on patterns of epitope targeting. The phylogenetic dependency network adds to the growing body of literature suggesting that sites of escape, order of escape, and compensatory mutations are largely consistent even across different clades, although we also identify several differences between clades. As recent case studies have demonstrated, understanding both the complexity and the consistency of immune escape has important implications for CTL-based vaccine design. Phylogenetic dependency networks represent a major step toward systematically expanding our understanding of CTL escape to diverse populations and whole viral genes.

## Author Summary

One of the enduring challenges facing HIV vaccine design is the remarkable rate of viral mutation and adaptation that limits the ability of the immune system to mount a lasting effective response. This rapid rate of mutation leads to extensive within- and between-host viral diversity that makes creation of a broadly reactive vaccine difficult. A first step in overcoming this challenge is to identify consistent patterns in viral adaptation. Recently, several studies have analyzed large groups of HIV-infected individuals and looked for correlations between HIV polymorphisms and the HLA class I alleles that restrict the cellular immune response. Here, we point out a limitation of previous approaches: correlations among HLA alleles and HIV codons lead to statistical confounding if not taken into consideration. In response, we develop two statistical models of evolution that explicitly represent stochastic selection pressure from multiple sources. After validating these models on synthetic data, we analyze the patterns of immune escape in a multicenter cohort of over 1000 individuals. Our results identify a dense network of interactions between HLA alleles and HIV codons, as well as among HIV codons, reflecting both a complexity and a promising consistency in the way that HIV adapts to the human immune response.

## Introduction

Cytotoxic T-lymphocytes (CTL) eliminate HIV-infected cells through the recognition of short virus-derived peptides, called *epitopes*, presented on the surface of the infected cell by Human Leukocyte Antigen (HLA) class I molecules [1]. The genes encoding the class I molecules are among the most polymorphic in the human genome, with each allele encoding a unique HLA molecule capable of presenting a variety of possible epitopes [2]. Due to rapid rates of mutation, HIV is able to evade the CTL response through the evolution of mutations in or around these epitopes that decrease antigen presentation and/or CTL recognition (CTL escape) [3]. Therefore, both the processes of antigen presentation to CTL as well as CTL escape are HLA-restricted. Indeed, CTL escape is to some extent predictable based on host HLA profile. That is, correlations between HLA alleles and HIV polymorphisms identified at the population level can identify important sites of immune selection on the viral genome and common pathways of immune escape [4],[5].

Moore et al. first demonstrated the presence of HLA footprints at the population level by identifying HIV polymorphisms that were associated with specific HLA alleles [6]. The 89 codons in the HIV protein Reverse Transcriptase that were reported to be correlated with HLA alleles suggested that CTL pressure was dramatically shaping HIV evolution. Moreover, the correlations reported by Moore et al. provided directly testable hypotheses about the specific escape pathways employed by HIV to evade the cellular immune response. Although their logistic regression analysis was later shown to lead to high false positive rates due to the confounding effects of the HIV phylogeny [7], the broad conclusions of the Moore et al. study were recently confirmed in two independent cohorts using methods that account for the phylogeny [8]–[11]. These population-based studies support the importance of the CTL response in the control of HIV.

This population approach can be considered an application of the *comparative method*, which is generally defined as the study of correlated evolution among traits (genotypes or phenotypes) or between traits and the environment [12]–[14]. In the classical case, the entities studied are species. In the case of HIV, the high rate of mutation leads to genetically distinct populations, called *quasispecies*, within each infected individual [15]–[17], making the two endeavors highly analogous.

Since the late 1970s, researchers have noted the confounding effect of phylogeny on the comparative method, with the classical approach for continuous traits provided by Felsenstein's method of independent contrasts [18] and a more general solution found in generalized least squares [19]. The problem of confounding arises due to the fact that some (quasi-) species are inherently more similar to each other than to other species by virtue of their relatively recent time since divergence. Thus, statistical analyses that assume samples are independent and identically distributed (such as those that use Fisher's exact test or logistic regression) have unexpectedly high variance and often exhibit systematic bias that increases both false positive and false negative rates [12], [18]–[21].

The comparative method for discrete traits has received much attention in the study of protein evolution. Here, the comparative method is used to identify coevolving codons within a protein or between proteins in the hopes of identifying structural or functional codon interactions and their resulting constraints on protein evolution (for review, see [22]). Although many methods that correct for phylogenetic structure have been proposed for this field [12], [20], [23]–[31], nearly all share a common weakness: computational considerations constrain the models to look for correlations only between pairs of attributes. Thus, where chains of interactions exist (*A*→*B*→*C*) these pairwise tests will fail to distinguish between *direct* associations (*A* — *B*) and *indirect* or, more specifically, *one-hop* associations (*A* — *C*), which may lead to incorrect hypotheses about the underlying biological system. These chains of interactions are almost certainly the norm in codon coevolution, as observed covariation is often driven by the constraints of three-dimensional physical interaction [32]–[36]. To date, only Poon et al. [37] have addressed chains of interactions in protein evolution in a phylogenetic context, although their method was not applied to the analysis of HLA-mediated selection pressure.

In the HIV field, the tasks of identifying codon covariation and HLA-mediated escape mutations have been treated as separate problems (see, e.g., [6]–[11],[23],[37]. If, however, both phenomena are widespread, then each will confound the other. When identifying HLA-mediated escape mutations, confounding due to codon covariation may arise in the case of compensatory mutations that partially reduce the fitness cost of the primary escape mutations [3], [38]–[41]. Because these escape mutations typically arise in the context of the compensatory mutations, both escape and compensatory mutations may appear correlated with the HLA allele in question. When identifying codon covariation, patterns of epitope targeting, including order of escape due to immunodominance and inter-patient variations in the overall strength of the immune system, will lead to patterns of correlation at the HIV codon level. In what follows, we show that these two processes significantly confound one another, making identification of codon covariation and HLA-mediated escape inextricably linked.

HLA linkage disequilibrium (LD) will further confound the detection of HLA-mediated escape mutations. Because the HLA class I loci are located in close proximity on chromosome 6, the alleles tend to be in tight LD, meaning inheritance of (e.g.) a specific HLA-B allele is strongly correlated with inheritance of a specific HLA-C allele [42]. Thus, a sufficiently powered study will tend to find both B and C alleles associated with each escape polymorphism, even if escape is driven by only one allele. The problem of HLA LD was first adjusted for in large scale HLA escape studies by Brumme et al. [8] and Rousseau et al. [10] who corrected for it by computing LD and assigning associations to alleles based on strength of the correlation as well as previously determined experimental evidence. More recently, Matthews et al. [11] presented an automated method for correcting for LD using the Decision Tree model, which we describe in Methods.

In summary, when detecting HIV escape mutations, there are at least two sources of confounding in addition to the phylogenetic structure of the HIV sequences: (1) covariation among HIV codons, and (2) HLA linkage disequilibrium. There is therefore a potential need for a statistical model that can accurately account for both sources of confounding in a phylogenetic context. In what follows, we describe a variation of the dependency network [43], what we call a *phylogenetic dependency network* (PDN), that accounts for phylogenetic relationships among the data by conditioning those relationships on a model of evolution. In addition, we describe the *Noisy Add* distribution, a parsimonious distribution (i.e., one with few parameters) that is suitable for modeling HIV escape and codon covariation. This Noisy Add distribution is a generalization of the distribution described by Carlson et al. [21], in which only pairwise correlations are considered.

We demonstrate the utility of phylogenetic dependency networks for modeling HLA-mediated escape in HIV. We first examine synthetic data generated from the Brumme et al. study [9] to demonstrate the need for simultaneously accounting for phylogenetic structure, HLA linkage disequilibrium, and HIV codon covariation, as well as to explore the ability of the PDN to detect these associations. We then extend the synthetic studies to examine method performance on datasets containing HIV sequences from two different subtypes (*clades*) and determine the power available for different cohort sizes. Finally, we undertake an HIV/HLA cross sectional analysis with the largest cohort to date, combining Gag p17/p24 sequences and individual HLA data from the HOMER cohort from British Columbia, Canada (predominantly HIV-1 clade B) [9] and the Durban cohort from Durban, South Africa (predominantly HIV-1 clade C) [10],[44] to yield a mixed clade B/C dataset of 1144 Gag p17/p24 sequences from chronically infected, HLA-typed, antiretroviral naïve individuals. Using this large cohort, we infer a dense phylogenetic dependency network that suggests a substantial role of HLA-mediated selection pressure in shaping HIV evolution along with specific predictions of escape pathways employed by the virus. Our model suggests that many patterns of escape are the same across the two clades. In addition, our model is consistent with known pathways of CTL escape and reveals many novel findings that should inform vaccine design.

## Methods

Here, we describe phylogenetic dependency networks generally and use the domain of HLA-mediated HIV codon evolution to illustrate the concepts. A dependency network represents the probabilistic dependencies among a set of predictor and target attributes. In our domain, *target attributes*, denoted **Y**, correspond to the presence or absence of amino acids at all codons in an HIV protein. For a given *Y* in **Y**, the *predictor attributes*, denoted **X**, correspond to the presence or absence of amino acids at all codons other than that for *Y* and the presence or absence of all HLA alleles. Note that all attributes are binary. We have found that this choice yields more statistical power in practice.

A dependency network (phylogenetically corrected or otherwise) has two components. The first component, sometimes referred to as the *structure of a dependency network*, is a directed graph linking nodes, where each node corresponds to one of the attributes in the domain. (We use the same name—e.g., *Y*—for the attribute and its corresponding node in the graph.) An arc from *X* to *Y* in the graph is a statement that the probability distribution for *Y* depends on *X*. Thus, in our domain, a dependency network graphically depicts which HLA and codon attributes predict each codon. The second component is a collection of conditional or *local* probability distributions, one for every target attribute of interest. The local probability distribution for target attribute *Y* is *P*(*Y*|**Xˆ**), where **Xˆ**⊆**X** are the parents of *Y* in the graph. Therefore, in our domain, a dependency network contains a probability distribution for each codon attribute conditioned on various HLA and codon attributes. When constructing a dependency network, each local probability distribution is learned independently. This approach is computationally efficient, although it can lead to a decrease in statistical efficiency (see Discussion).

**Figure 1:**
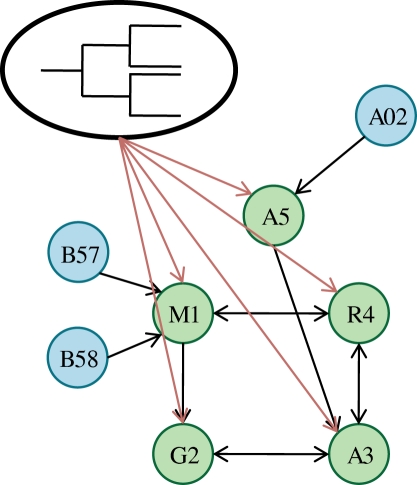
Phylogenetic dependency network (PDN). A PDN is a graphical model consisting of target attributes whose outcome is a probabilistic function of predictor attributes. Each of these probabilistic functions takes the phylogeny of the sequences into account. Here, the target attributes (green nodes) are binary and represent the presence or absence of amino acids at codons. These target attributes may have dependencies on other codons (codon covariation) and/or on HLA alleles (HLA-mediated escape), which are denoted by blue nodes. Arcs represent the learned dependencies between target and predictor attributes. All target attributes are assumed to be influenced by the phylogeny (red arcs). The probability components of a PDN are the local conditional probabilities, each of which relates a single target attribute to the phylogeny and a subset of the predictor attributes. These local conditional probabilities are learned independently for each target attribute. In the hypothetical example depicted here, B*57 and B*58 predict M1 and A*02 predicts A5. A5 predicts A3, and there is a cyclical dependency among M1, G2, A3 and R4, in which most of the arcs are bidirectional.

A phylogenetic dependency network (PDN) for our HIV application is a dependency network in which each local probability distribution is corrected for the phylogenetic structure of the HIV sequences. That is, the probability that a codon in an individual is a given amino acid depends on not only the attributes **Xˆ**, but also on where that individual's HIV sequence sits in the phylogeny (Figure 1). Specifically, a PDN is a collection of the distributions *P*_Ψ_(*Y*|**Xˆ**), one for each *Y* in **Y**, where *P*_Ψ_ refers to a distribution corrected for phylogeny.

In this paper, we use a model-selection approach to identify **Xˆ**, the set of parents for *Y*. Specifically, we use significance tests—False Discovery Rate (FDR) thresholds based on likelihood-ratio tests (LRTs)—to determine **Xˆ**. To avoid the inappropriate use of an LRT, we exclude attributes as possible predictors when the corresponding predictor-target pair has a 2×2 contingency table that includes at least one bin where both the observed and expected value is at most three. This parameter was chosen based on performance with independent data (not shown).

### Phylogenetically Corrected Distributions for One Predictor Attribute

A simple approach for identifying a set of attributes that predict a given codon (i.e., for identifying the parents of a target attribute in a PDN) is to test for pairwise correlations between a target codon and each predictor attribute. The details of a statistical model that follows this approach, hereafter referred to as the *univariate model*, are described in the section “Model Details” and evaluated in [21]. We will review the univariate model here, as it forms a basis for our multivariate model.

**Figure 2:**
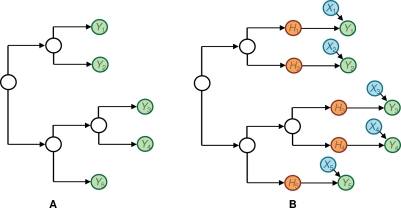
The univariate model. (A) The null model, in which an amino acid evolves independently down the tree until it reaches a leaf. (B) The alternate model, in which an amino acid evolves independently down the tree until is reaches an individual, where it is influenced by selection pressure from the predictor. The variable *H_i_* for the *i*th individual represents the variable *Y_i_* had there been no influence from *X_i_*. Only the *Y_i_* and *X_i_* are observed. Conditional probability distributions are not shown.

To determine whether there is a significant pairwise correlation between predictor attribute *X* and target amino acid *Y*, we compare the likelihood of a null model that reflects the assertion that *Y* is under no selection pressure to an alternative model that reflects the assertion that *Y* is under selection pressure induced by a single predictor attribute *X*. The null model assumes the target codon *Y* can be described completely by a model of independent evolution along a phylogenetic tree (Figure 2A). The leaves of the tree correspond to individuals in the study and are typically observed. The interior nodes of the tree correspond to unseen individuals infected by an HIV sequence that is a point of divergence. These nodes are hidden—that is, never observed. We use *Y_i_* to denote attribute *Y* for the *i*th individual in the study (*i* = 1,…, *N*). (Note that *Y_i_* is a *variable* in the ordinary statistical sense.) Because each target attribute is binary, a natural null model is the two-state version of the continuous time Markov process, commonly used in phylogenetics [45]. This model assumes evolution is independent between different branches of the phylogeny and that the only informative predictor of a node in the evolutionary tree is its parent node in the tree.

The alternative model adds a component of selection pressure derived from the predictor attribute *X* (Figure 2B). We use variable *X_i_* to denote the attribute *X* for the *i*th individual in the study (*i* = 1,…, *N*), and do not explicitly name *X* for the unseen individuals represented in the interior of the phylogenetic tree. Because *X* may not share the same evolutionary history as *Y*, we assume *X* influences *Y* only at the leaves of the tree. In particular, we assume that, among the variables corresponding to attribute *X*, only *X_i_* influences *Y_i_* for each *i*. This assumption was evaluated more fully by Carlson et al. [21] and found to be a reasonable approximation, even when *X* and *Y* share the same evolutionary history. To model selection pressure at the leaves, we extend the null model by adding a hidden attribute *H* (with corresponding variables *H_i_*, *i* = 1,…, *H*) that represents what *Y* would have been had there been no selection pressure. The probability distribution for *Y_i_* then depends on *H_i_* and *X_i_*. When the values of *H_i_* and *Y_i_* are different, we say that a transition conditioned on *X_i_* has taken place. The precise rules governing the transitions conditioned on *X_i_* are given by the *univariate leaf distribution P*_Ψ_(*Y*|*X*) = *P*(*Y_i_*|*H_i_*, *X_i_*). We assume that this leaf distribution is not a function of *i*—that is, this distribution is the same for each individual *i* = 1,…, *N*. Also note that the subscript Ψ is a reminder that *Y_i_* depends not only on *X_i_*, but also on the phylogeny through variable *H_i_*.

In the univariate case, we define four possible leaf distributions. *Escape* means an individual may transition to *Y_i_* = 0 only when *X_i_* = 1. *Reversion* means an individual may transition to *Y_i_* = 1 only when *X_i_* = 0. *Attraction* means an individual may transition to *Y_i_* = 1 only when *X_i_* = 1. *Repulsion* means an individual may transition to *Y_i_* = 0 only when *X_i_* = 0. Given a univariate leaf distribution, a single parameter *s* specifies the probability that the transition occurs given the appropriate state of *X_i_*. Note that attraction/repulsion correspond to a positive correlation between *X_i_* and *Y_i_*, whereas escape/reversion correspond to a negative correlation.

The names of these leaf distributions correspond to various processes for selection pressure [21]. For example, the B*57-restricted CTL response selects for escape from the *susceptible* threonine at position 242 of the HIV Gag protein [46]. So, from the perspective of hidden and target attributes that correspond to the presence and absence of threonine, the amino acid can transition from threonine to not threonine (*H* = 1, *Y* = 0) with a non-zero probability only when the individual has the B*57 allele (*X* = 1), which corresponds to the escape distribution just described. In addition, escape from threonine bears a fitness cost that leads to reversion in B*57-negative individuals [46]. Consequently, the amino acid can transition from not threonine to threonine (*H* = 0, *Y* = 1) with non-zero probability only when the individual lacks B*57 (*X* = 0), corresponding to the reversion distribution. The codon for threonine usually escapes to the *resistant* amino acid asparagine [46]. Continuing the example from the perspective of hidden and target attributes that correspond to the presence and absence of asparagine, the amino acid can transition from not asparagine to asparagine (*H* = 0, *Y* = 1) only when the individual has the B*57 allele (*X* = 1), which corresponds to the attraction distribution. Finally, the amino acid can transition from asparagine to not asparagine (*H* = 1, *Y* = 0) only when the individual lacks the B*57 allele (*X* = 0), which corresponds to the repulsion distribution. Although there is a natural pairing between escape/reversion and attraction/repulsion, in that the former indicates a negative correlation and the latter a positive correlation, the processes are each distinct and may provide information as to the underlying mechanism (see the section on distinguishing leaf distributions in Results). Furthermore, whereas the vast majority of clinically-derived HIV sequences have either threonine or asparagine at codon 242, most codons are more variable, with more than one amino acid susceptible to, or resistant from, CTL pressure mediated by the HLA allele. Consequently, escape/attraction and reversion/repulsion for alternate amino acids often provide additional information. Note that by restricting the univariate leaf distribution to one of these four forms, we have assumed that only one process (escape, reversion, attraction, or repulsion) is occurring for a given predictor-target pair. Although in reality both escape and reversion (or attraction and repulsion) may occur with the same HLA-epitope combination, relaxing our assumption leads to substantial loss of power. Thus, we apply each of the four leaf distributions to the predictor-target pair and include only the most significant correlation in the model.

### Phylogenetically Corrected Distributions for More Than One Predictor Attribute

**Figure 3:**
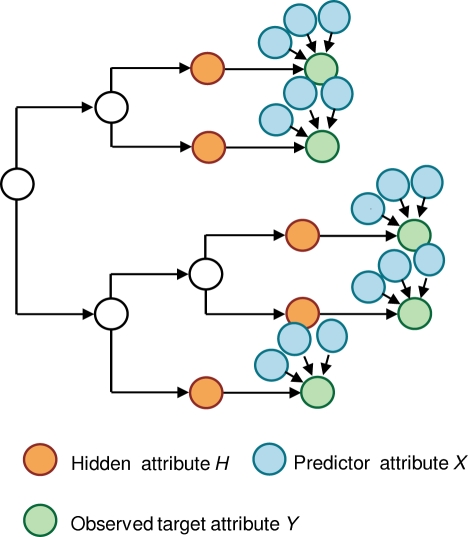
The multivariate model. Here, an amino acid evolves independently down the tree until is reaches an individual, where it is influenced by one or more predictor attributes.

The univariate model works well when there are no correlations among predictor attributes or among target attributes [21]. As discussed, however, use of the model in the presence of linkage disequilibrium among HLA alleles and HIV codon covariation will likely lead to spurious associations. To avoid this problem, we use a *multivariate model*, in which more than one attribute can be used to predict a particular target attribute. In this model, for a given target attribute *Y*, shown in Figure 3, the target attribute is allowed to evolve independently down the tree until it reaches a leaf in the tree corresponding to an individual in the study. At this point, selection pressure within the individual is governed by a *multivariate leaf distribution*, denoted *P*_Ψ_(*Y*|**Xˆ**), which depends on multiple predictor attributes **Xˆ**. As in the univariate case, this leaf distribution is the same for each individual *i* = 1,…, *N*.

The set of significant predictor attributes can be identified by a number of methods including forward, backward, and forward/backward selection. In this work, we concentrate on forward selection, wherein **Xˆ** is iteratively augmented with the most significantly associated attribute at each iteration. For each added attribute, we record only the most significant leaf distribution (escape, reversion, attraction, or repulsion). The significance of a predictor *X* with respect to target attribute *Y* is computed using false discovery rates based on an LRT in which both the null and alternative models are conditioned on all significant predictors that were identified in previous iterations of forward selection. For practical purposes, we terminate forward selection when the most significant association has a *p*-value greater than or equal to some threshold to be described.

There any many possibilities for the form of the multivariate leaf distribution *P*_Ψ_(*Y*|**Xˆ**). In this paper, we consider two distributions: Decision Tree and Noisy Add.

#### Decision Tree

A straightforward way to represent the multivariate leaf distribution *P*_Ψ_(*Y*|**Xˆ**) is to list the probability distribution for *Y* given every possible instance of the attributes *H* and **Xˆ**. Unfortunately, the length of this list grows exponentially with the number of predictor attributes. An alternative is to use a Decision Tree, which is a compact representation of such a list. The use of the Decision Tree as a multivariate leaf distribution was recently employed by Matthews et al. [11] to account for HLA LD. Here, we describe the approach in some detail.

**Figure 4:**
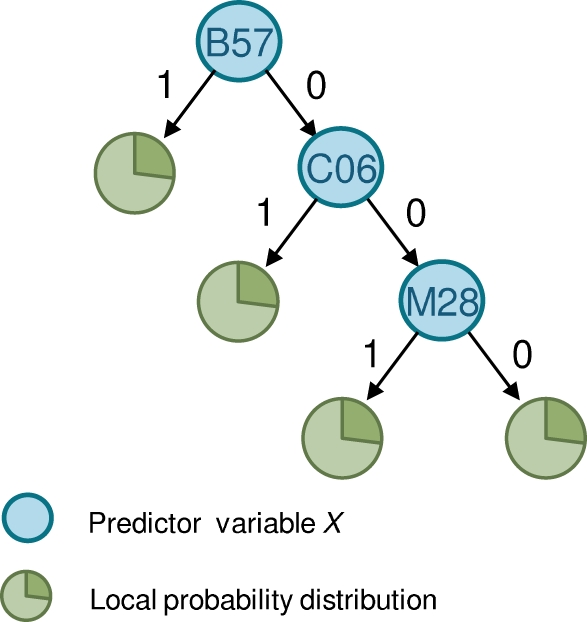
Decision Tree leaf distribution. Each path from root to leaf yields a distinct local probability distribution.

A graphical depiction of the Decision Tree leaf distribution is shown in Figure 4. Note that this tree should not be confused with the phylogenetic tree. To help avoid this confusion, we use the term *tip* to refer to the bottom points on the Decision Tree. Each path in the tree from root to tip defines a particular instance of a subset of the attributes **Xˆ**, which in turn defines a conditioning event for the distribution of the target attribute. For example, in Figure 4, we consider the set of predictor attributes **Xˆ** = (B57, C06, M28), with each branch labeled 0 or 1. The path that follows the value 0 for the attribute B57, the value 0 for the attribute C06, and the value 1 for the attribute M28 corresponds to the instance (B57 = 0, C06 = 0, M28 = 1)—that is, the individual has M28 but not B57 or C06. At the tip of this path sits the corresponding conditional probability distribution *P*_Ψ_(*Y_i_*|B57 = 0, C06 = 0, M28 = 1). In general, each tip *k* in the Decision Tree is associated with the conditional distribution *P*_Ψ_(*Y_i_*|**Xˆ** = path*_k_*), where path*_k_* is the conditioning event corresponding to the *k*th path. The collection of these conditional distributions over all tips constitutes the multivariate leaf distribution.

A Decision Tree leaf distribution can be constructed in many ways. As mentioned, we use forward greedy search. First, we initialize the tree to a single root node, which is simply the univariate leaf distribution for the most significant attribute. We then grow the tree iteratively. At each iteration, we consider extending (or *splitting*) a tip node *k* on some attribute not already in the path to the tip. When splitting tip node *k* on an attribute *X*, the node is replaced with two branches and two corresponding tip nodes. The left and right branches correspond to adding *X* = 1 and *X* = 0, respectively, to the conditioning event associated with the original tip node. The split is made if the resulting local distribution is a significantly better estimate than that prior to the split, as measured by an LRT. The LRT is computed using the univariate model applied to those individuals whose attribute values match those described by path*_k_*. To make the process more efficient in our HIV application, we consider splitting the tip node only under the path *X* = 0 for all *X* in **Xˆ**. That is, we repeatedly apply the univariate model to all individuals for whom *X* = 0 for all the previously identified significant predictor attributes. We iterate this process until no significant predictors are found, using a threshold of *p*<0.05.

#### Noisy Add

One drawback of the Decision Tree approach is that, as the tree grows, the number of samples that we use to test for the next split decreases. Rather than consider smaller and smaller subsets of the data, the Noisy Add leaf distribution models selection pressure as an additive process among the predictor attributes. That is, the Noisy Add leaf distribution is based on the assumption that each predictor attribute independently contributes a positive or negative selection pressure on the target attribute. These pressures then sum to determine the value of the target attribute.

In the univariate case, each leaf distribution can be seen as representing three mutually exclusive and exhaustive events (for each individual): (1) the selection pressure is absent, either because the state of the predictor attribute excludes selection pressure or, with probability 1−*s*, no transition occurred despite the potential for selection pressure; (2) selection pressure leads to *Y_i_* = 1 (attraction or reversion); or (3) selection pressure leads to *Y_i_* = 0 (escape or repulsion). We can represent these three possible events by a hidden attribute *I* that takes on the values 0, 1, and −1, respectively. Given a set of *M* predictor attributes, we can associate a hidden variable 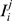 for the *j*th attribute in the *i*th individual. Then, assuming that selection pressure across the predictor attributes contributes independently and equally to the outcome of *Y_i_*, we can determine the outcome of *Y_i_* by summing the values of the 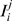 variables: 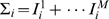. If Σ*_i_* is 0, then it is as if no selection occurred. If Σ*_i_*<0, then negative selection (escape/repulsion) has occurred, and the target variable *Y_i_* will be zero. If Σ*_i_*>0, then positive selection (attraction/reversion) has occurred, and the target variable will be one. Of course, we don't know the actual values of *I^j^* for each predictor variable, so we must sum over the possibilities, resulting in a probability distribution over Σ*_i_*. The strength or frequency of selection pressure contributed by each predictor attribute *j* is captured by the parameter *s^j^*. Like the corresponding parameter *s* in the univariate model, *s^j^* is the probability that the predictor attribute exerts selection pressure (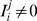), given the appropriate state for the predictor attribute. A more precise definition of Noisy Add, including the generalization from the univariate model, specifics of learning the parameters *s^j^*, and methods for reducing computation time can be found in the section on model details.

The contribution of a given predictor attribute *X^j^*∈**Xˆ** as a predictor of target *Y* is quantified using an LRT against the null model consisting of **Xˆ**−*X^j^*. The most significant predictor attribute is added to the Noisy Add model on each iteration, stopping when the most significant predictor fails to achieve *p*<0.005. (We use a more aggressive threshold than that for Decision Tree because Noisy Add is more computationally intensive.)

### *q*-Values

We identify significance using *q*-values [47], which conservatively estimate the false discovery rate (FDR) [48] for each *p*-value. The FDR is defined to be the expected proportion of false positives among results called significant at a given threshold *t*. The *q*-value of *t* is the minimum FDR observed for all *t*′≥*t* [47]. Following Storey and Tibshirani [47], we use the approximation
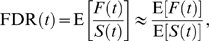
(1)where *S*(*t*) is the number of associations called significant at *t* and *F*(*t*) is the number of true nulls (false positives) at *t*. To estimate the numerator, we order the *p*-values of the association tests in increasing order *p*_1_,…, *p_m_* and use the approximation E[*S*(*p_i_*)]≈*S*(*p_i_*) = *i*. To compute E[*F*(*t*)], Storey and Tibshirani point out that uniformity of *p*-values allows the approximation
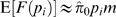
(2)where 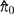 is a (conservative) estimate of the proportion of all hypotheses that are truly null. In our case, we assume *a priori* that the vast majority of the many hypotheses tested will be null (i.e., most codons and HLA alleles have no direct effect on a given target attribute), and so conservatively set 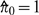.

**Figure 5:**
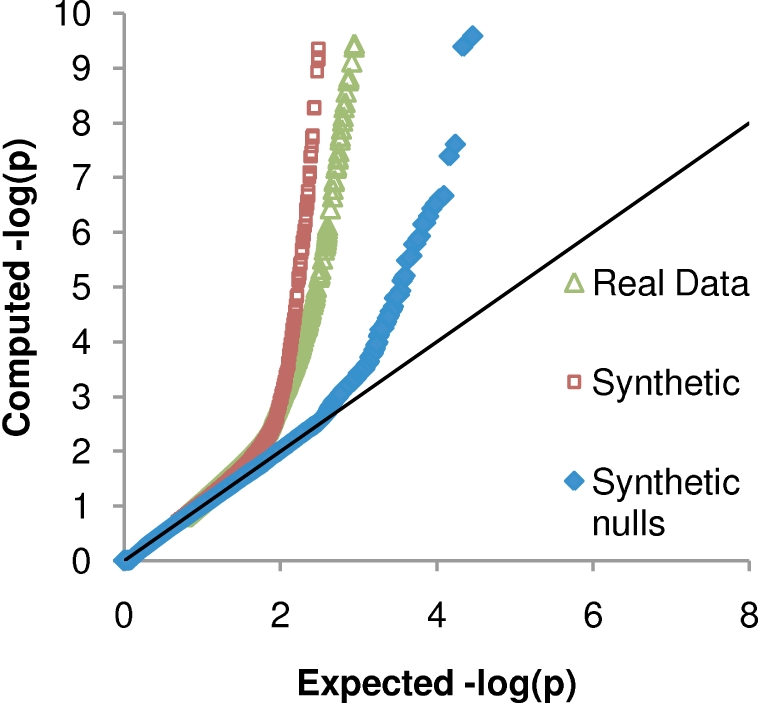
Quantile-Quantile (QQ) plot of *p*-values on the mixed clade cohort. Values correspond to −log_10_(*p*).

The asymptotic conservative guarantee of (1) requires a conservative estimate of (2), which requires a valid (or stochastically conservative) *p*-value. In order to achieve a valid *p*-value, all model assumptions must be reasonably met. In particular, all sources of confounding must be accounted for. In principle, our multivariate models can account for these sources, provided the input phylogeny is reasonable and all other sources of confounding are provided as predictor attributes in **Xˆ**. To confirm this argument for the Noisy Add leaf distribution, we constructed QQ plots using the mixed clade dataset and a synthetic dataset as described in the following section (Figure 5). On null synthetic data (i.e., synthetic data in which no predictor-target pairs where associated), the QQ plot indicates that Noisy Add yields a uniform distribution of *p*-values. The distribution starts deviating from expected at *p*<0.001, at which erroneous associations that represent one-hop associations and associations with wrong arc direction start skewing the distribution. Including a panel of 550 synthetically planted non-null associations (see the following section) shifts the distribution as expected. Likewise, the distribution on real data follows the pattern observed on synthetic data that includes planted associations.

Alternatively, E[*F*(*t*)] can be estimated from null data by (e.g.) permuting the predictor attributes [21],[49], though permutation breaks any covariation among predictors and may lead to biased estimates.

In what follows, we compute *q*-values using the method of Storey and Tibshirani for Noisy Add for the model that corrects for phylogeny, LD, and covariation, and the permutation test for all other models, as these models fail to account for key sources of confounding, rendering their *p*-values stochastically liberal. For these other models, we found that across all datasets tested, the permutation test was more conservative than using equation (2), which consistently yielded overly liberal *q*-values (data not shown).

Finally, FDR represents the expected proportion of tests called significant that are null. When different classes of predictor attributes are considered, this may lead to confusion. For example, an FDR of 20% for an association between an HLA allele and codon does not imply that 20% of *HLA-codon* associations at that *p*-value are expected to be null, but that 20% of *all* associations (including codon-codon associations) are expected to be null. Thus, we find it useful to compute FDR separately for each class of association. In what follows, we compute *q*-values for HLA-codon and codon-codon associations separately. The final combined association lists are then ordered by the *q*-values, with ties broken by *p*-values.

### Data

These methods were applied to population-based HIV sequences from chronically infected, antiretroviral naïve and HLA-typed individuals from two cohorts: the *HOMER* cohort from British Columbia, Canada, consisting of 567 predominantly clade B gag sequences [9], and the *Durban* cohort, consisting of 522 predominantly clade C p17/p24 gag sequences from Durban, South Africa [10],[44]. Individuals in the HOMER and Durban cohorts were HLA-typed to two- and four-digit resolution, respectively. Here, we truncate the Durban data to two-digits for comparison with the HOMER cohort. Viral sequences were determined by nested reverse-transcriptase polymerase chain reaction (RT-PCR) amplification of extracted plasma HIV RNA followed by bulk sequencing, as previously described [8]–[10]. Phylogenies were constructed from these sequences using PHYML [50], run using the general time reversible model over the HIV sequences and estimating all parameters via maximum likelihood.

Synthetic datasets were designed to mimic the real datasets as closely as possible. We first fit a specified model to the real data to identify parameters and *q*-values for each predictor-target pair. We then planted predictor-target pairs for each significant (*q*≤0.2) predictor-target pair identified from the real data. Specifically, we generated a synthetic target amino acid for each consensus amino acid in the sequence, such that (1) if the amino acid had no significant (*q*≤0.2) associations, then the amino acid was generated according to the parameters of the independent evolution model (the null model from the univariate case), and (2) if the amino acid had *M*>0 associations, then the amino acid was generated according to the given multivariate model with the predictor parameters *s* ^1^,…, *s^M^*, taken from the real data. When an observation was missing in the real data, the corresponding observation in the synthetic data was also made to be missing. We treated amino acid insertions/deletions and mixtures as missing data.

Our goal was to generate data that is as realistic as possible, both in the values of the parameters used and the number of predictors deemed correlated with the target. Because our recall rate is less than 100% (see section on synthetic results), planting only those associations that are found in the real data would result in a smaller proportion of synthetic predictor-target pairs called significant than real predictor-target pairs called significant. We therefore planted two associations for every observed significant association in the real data and reduced the number of independently evolving codons accordingly. For the Noisy Add model, this procedure planted 72 HLA-codon and 612 codon-codon associations in the HOMER cohort and 114 HLA-codon and 952 codon-codon associations in the combined HOMER-Durban cohort. In hindsight, doubling the number of planted associations was an overcompensation, as experiments on this synthetic data yielded a 75% recall rate. Nonetheless, the doubling produced a reasonable result, as Noisy Add declared 0.56% of all synthetic predictor-target pairs significant at *q*≤0.2 compared to 0.65% of all predictor-target pairs in the real data for the combined HOMER-Durban cohort.

### Data Analysis

As mentioned, we binarized all data. For example, if three amino acids were observed at a given sequence position, we created three binary attributes corresponding to the presence and absence of each amino acid. When reporting results, however, we assumed that the most relevant information was at the codon level. Thus, unless stated otherwise, *HLA-codon* associations refer to the most significant associations between an HLA allele and any observed amino acid at the codon under any of the four leaf distributions. Likewise, *codon-codon* associations refer to the most significant association between the codons over all the associations computed for the complete repertoire of observed amino acids and possible leaf distributions at those codons. This approach was taken exclusively in the synthetic studies, though the results were similar when we looked at exact associations (at the level of observed residues and leaf distributions; data not shown).

We report power results as Precision-Recall (PR) curves, where the x-axis is *recall* (*TP*/(*FN*+*TP*)) and the y-axis is *precision* (*TP*/(*TP*+*FP*)), where TP is the number of true positives, FP is the number of false positives, and FN is the number of false negatives. To construct PR curves, we computed precision and recall for every observed *q*-value for each method. We used as a gold standard the synthetic data as described in the previous section. Accuracy of *q*-values, called *calibration*, is plotted as (1−Precision) versus *q*-value. A perfectly calibrated result is a line with slope one. To compare two PR curves, we computed *p*-values using the absolute value of the difference between the areas under the two curves as the statistic. The null distribution assumes the two curves will on average provide the same ranking over the predictor target pairs and is constructed using a permutation test in which two pseudo-curves are generated by randomly swapping the ranks between the two methods for each predictor-target pair. That is, if methods 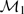 and 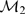 provide ranks of *r*_1_ and *r*_2_, respectively, for a predictor target pair *PT*, then with probability 0.5, 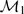 will be reassigned rank *r*_2_ and 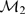 will be reassigned rank *r*_1_ for *PT*. Resulting ties in ranks were broken at random. Ranks were used rather than *q*-values so that the scores of two uncalibrated methods could be compared directly. 10,000 permutation tests were run to compute each *p*-value.

When we refer to associations involving codons, we will sometimes find the following notation useful. T242 will refer to an amino acid (in this case threonine) at a specific codon (242). If the association is escape or reversion, then T242 is the *susceptible* form. If the association is attraction or repulsion, then T242 is the *resistant* form. The PDN will often find complementary associations. For example, T242 is the susceptible form with respect to B*57, and N242 is the resistant form. We will sometimes refer to such associations as T242N. For simplicity, we will usually report only the smaller *q*-value of the two associations. If only the susceptible association is significant (*q*≤0.2), we will sometimes write T242X. Likewise, if only the resistant is significant, we will sometimes write X242N.

Optimally defined epitopes [51] were taken from http://www.hiv.lanl.gov/content/immunology/tables/optimal_ctl_summary.html, accessed on December 21, 2007. To allow the inclusion of processing mutations [52], we called an association a match to the optimal epitope if it was within three codons of the epitope boundary, as described elsewhere [8],[10]. When using the optimal epitopes as a bronze standard for comparing methods, we considered only the most significant HLA-codon association per HLA-epitope pair to prevent double counting that arises due to the extent of within-epitope covariation (see Results). Similarly, in cases where an HLA-codon pair was not within three codons of an optimal epitope, we computed the most likely predicted epitope using Epipred [53] so that neighboring associations in putative epitopes were not double counted.

### Model Details

In this section, we provide details regarding the univariate and Noisy Add models, in addition to a brief discussion on computational requirements for the models.

#### Details of univariate model

First, let us consider the null model. Consider target attribute *Y* that denotes the presence (*Y* = 1) or absence (*Y* = 0) of a particular amino acid at a particular codon. We use variable *Y_i_*, *i* = 1,…, *N* to denote the attribute *Y* for the *i*th individual in the study. (We use corresponding notation for predictor attributes and variables.) It is quite common to assume that the variables *Y*_1_,…, *Y_N_* are independent and identically distributed (IID). In our application, however, the variables are related through a phylogenetic tree. We can model these relationships using a probabilistic phylogenetic model as shown in Figure 2A. Nodes at the leaves of the tree, labeled *Y*_1_,…, *Y_N_* correspond to the variables with the same name. (In general, we will use the same designation for both a variable and its node.) Unlabeled nodes in the interior of the tree correspond to events of divergence. We use Ψ to denote the structure (branchings and branch lengths) of the tree.

Associated with each variable (or node) *B* in this phylogenetic tree is a conditional probability distribution *P*(*B*|*A*), where *A* is the parent node of *B*. As in the probabilistic model of Felsenstein [45] for a phylogenetic tree, we assume that the conditional probability table is described by a continuous time Markov process (CTMP) and parameterized by *θ* = (*π*,*λ*), where *π* is the stationary distribution of *Y* = 1 and *λ* is the rate of mutation. The conditional probability table of the CTMP from parent node *A* to child node *B* along a branch of length *d* is given by
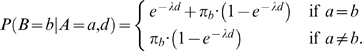
(3)where *π_b_* = *π* when *b* = 1, and *π_b_* = 1−*π* when *b* = 0. This evolution model is reversible, making the choice of root in the tree arbitrary [45].

Given a set of observations for (typically, all of) *Y*_1_,…, *Y_N_*, there are several criteria that can be used to identify good values for the parameters *π* and *λ* and the structure Ψ of this model (or, in the Bayesian case, a distribution over these quantities). For this and all models discussed in this paper, we choose parameters and structure using the maximum likelihood criterion, as is done in (e.g.) [45]. There are a number of methods for identifying the maximum-likelihood parameters, including gradient decent and the Expectation-Maximization (EM) algorithm. In this paper, we use the EM algorithm [54] to learn *θ*. To learn the structure Ψ, we apply PhyML to the (gag p17–p24) nucleotide sequences using the general time reversible GTR model with all other parameters estimated from the data [50].

We denote this null model *P*_Ψ_(*Y*|*θ*), as it represents a phylogenetically corrected distribution for *Y*. Note that this model includes the situation where the observations of *Y*_1_,…, *Y_N_* are IID as a special case (i.e., the limit as *λ* tends to infinity.)

Now let us consider the alternative model, which reflects the assumption that a codon is under selection pressure induced by a single predictor attribute *X*. To construct this model, shown in Figure 2B, we begin with the null model and first change each *Y_i_* to *H_i_*, which represents what *Y_i_* would have been had there been no influence from *X_i_*. Then, we assume that, for each individual *i*, the probability distribution for *Y_i_* depends on *X_i_* and *H_i_*. Further, we assume that these conditional distributions *P*(*Y_i_*|*H_i_*, *X_i_*) are the same for each individual *i*, and collectively denote them by *P_Ψ_*(*Y*|*X*). In general, this *univariate leaf distribution* can have four parameters corresponding to the four states of the conditional variables *H_i_* and *X_i_*. In our experience, however, use of such a distribution leads to loss of power. Consequently, we consider four separate distributions (as was previously defined [21]) and, for any given association, choose the one that best fits the data:

**Escape** *P*(*Y_i_* = 0|*H_i_* = 1, *X_i_* = 1) = *s*>0; *P*(*Y_i_* = 1|*H_i_* = 0, *X_i_* = 1) = 0; *P*(*Y_i_* = *a*|*H_i_* = *a*, *X_i_* = 0) = 1. That is, *H_i_* and *Y_i_* can be in different states only when *H_i_* = 1 and *X_i_* = 1.

**Reversion** *P*(*Y_i_* = 1|*H_i_* = 0, *X_i_* = 0) = *s*>0; *P*(*Y_i_* = 0|*H_i_* = 1, *X_i_* = 0) = 0; *P*(*Y_i_* = *a*|*H_i_* = *a*, *X_i_* = 1) = 1. That is, *H_i_* and *Y_i_* can be in different states only when *H_i_* = 0 and *X_i_* = 0.

**Attraction** *P*(*Y_i_* = 1|*H_i_* = 0, *X_i_* = 1) = *s*>0; *P*(*Y_i_* = 0|*H_i_* = 1, *X_i_* = 1) = 0; *P*(*Y_i_* = *a*|*H_i_* = *a*, *X_i_* = 0) = 1. That is, *H_i_* and *Y_i_* can be in different states only when *H_i_* = 0 and *X_i_* = 1.

**Repulsion** *P*(*Y_i_* = 0|*H_i_* = 1, *X_i_* = 0) = *s*>0; *P*(*Y_i_* = 1|*H_i_* = 0, *X_i_* = 0) = 0; *P*(*Y_i_* = *a*|*H_i_* = *a*, *X_i_* = 1) = 1. That is, *H_i_* and *Y_i_* can be in different states only when *H_i_* = 1 and *X_i_* = 0.

This model is reversible in the sense that the choice of root node among non-leaf nodes does not affect the likelihood of the data. We also note that, in principle, all parameters *θ* = (*π*, *λ*, *s*) and the structure Ψ can be optimized simultaneously. In practice, however, we find that using the structure Ψ learned in the absence of information about *X* works well, and is computationally more efficient. In addition, it may seem counter-intuitive that the HLA alleles of the individuals corresponding to the interior nodes of the phylogeny are not being taken into account. A path from one node to the next in the phylogeny, however, presumably reflects a series of infections over many individuals, some who will have the allele and some who will not. Thus, there will be some net evolution, which we account for by optimizing the parameters *π* and *λ* for each codon individually. Finally, we note that this model can be thought of as a (discrete) mixed-effects model, wherein the predictor variables *X_i_* correspond to the fixed effects and the hidden variables *H_i_* correspond to the random effects [55]. Rather than being related by (e.g.) a Gaussian covariance matrix, the random effects are related by a phylogenetic tree.

Both the null and alternative models are instances of what is known as a *generative* or *directed acyclic graphical* (DAG) model. In general, a generative model consists of a structure, a directed acyclic graph, in which nodes correspond to variables and missing arcs specify conditional independencies among the variables, and a set of conditional probability distributions, one distribution for each node. The conditional probability distribution for a given node is the distribution of the node given its parents. The conditional independences specified by the structure of the graph allow the joint distribution of the data to be written as the product over the nodes of their conditional distributions. The independences represented by the model facilitate computationally efficient inference, parameter estimation, and structure learning [56]. Importantly, given a set of parameters learned from real data, synthetic data can be easily generated from the model. When constructing PDNs, we separately learn a DAG model to encode each local probability distribution. As mentioned in the Discussion, however, one can restrict the arcs in a PDN to be acyclic, thus resulting in a single (phylogenetic) DAG model for all the attributes in the dataset.

In the following section, we consider the multiple-predictor case and again use graphical models to represent phylogenetically corrected distributions. As we shall see, the computational efficiencies afforded by graphical models will play an even more important role.

#### Details of Noisy Add model

**Figure 6:**
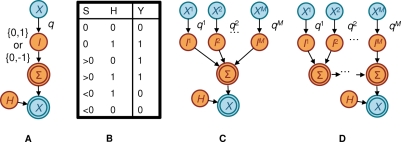
Noisy Add leaf distribution. (A) A generative process for the univariate leaf distribution. Here, the hidden variable *I* takes on a value of 0, 1 or −1 depending on whether selection pressure is absent, positive, or negative. (The subscript *i*, denoting a particular individual, is suppressed for simplicity.) The result is copied to Σ, which determines the result of the selection pressure. (B) The function that maps Σ and *H* to *Y*. (C) A generative process for the multivariate Noisy Add leaf distribution. (D) The grouping of the multivariate Noisy Add leaf distribution into a series of summations, grouped as Σ_2_ = *I*_1_+*I*_2_, Σ_3_ = Σ_2_+*I*_3_, and so on. This grouping makes inference much faster.

To understand the Noisy Add leaf distribution, let us recast the univariate distribution as the generative process shown in Figure 6A. (Recall that this distribution is independent of *i*. In the text that follows, we describe this and the generalized process for an arbitrary individual *i*. In the corresponding figures, we drop the subscript *i* to simplify the notation.) If *X_i_* = 1 (for escape or attraction; *X_i_* = 0 for reversion or repulsion), a coin weighted with probability *s* for heads is flipped. If the coin lands heads, then the intermediate variable *I_i_* gets the value 1 (for attraction or repulsion; −1 for escape and reversion). Otherwise, *I_i_* gets the value 0, corresponding to no selection pressure. The value of *I_i_* is then copied to the value of another variable Σ*_i_*. (The copy is not necessary here, but will help us generalize.) Finally, the target variable *Y_i_* is assigned a value based on the deterministic function shown in Figure 6B. With a little checking, it can be seen that this process produces precisely the univariate leaf distributions for escape, reversion, attraction, and repulsion.

The generalization of this process to multiple predictor variables 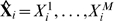 is shown in Figure 6C. Here, there is an 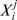 and 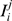 node for each predictor variable 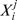. The weight on the coin is possibly different for each predictor variable. We use *s^j^* to denote the weight for predictor variable 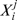, and *s* to represent the collection of parameters (*s* ^1^,…, *s^M^*). The variable Σ*_i_* is now a sum of the intermediate variables 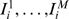. Finally, as in the univariate case, *Y_i_* is a deterministic function of Σ*_i_* and *H_i_* as given in Figure 6B.

Applying this generative process to individuals *i* = 1,…, *N*, we obtain the conditional distribution 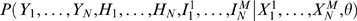, where *θ* = (**s**,*π*,*λ*) are the parameters of the model. Maximum likelihood values for these parameters can be inferred efficiently. The summation 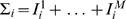 can be grouped as 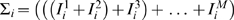, yielding the graphical model shown in Figure 6D. This grouping makes it possible to compute the distribution for *Y_i_* for any instance of the variables **Xˆ** *_i_* and *H_i_* in time that is quadratic in *M*. Furthermore, given any instance of the predictor variables *Xˆ* *_i_*, *H_i_*, and *Y_i_*, the probability distributions for 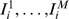 can be computed in time that is quadratic in *M*. Consequently, we can use the EM algorithm to estimate the parameters **s** efficiently. To estimate the full set of Noisy Add parameters *θ*, we embed this estimation procedure within an outer loop as follows.

#### E-step

Compute 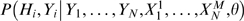 using any standard algorithm for graphical models (e.g., [56]).

Iterate to convergence in likelihood:

#### *E-step*

**For** *j* = 1,…, *M*

Compute
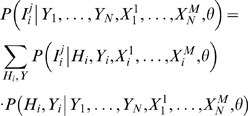
(4)


#### *M-step*

Given these probabilities, choose *s* to maximize the likelihood

#### M-step

Given these probabilities, choose *π* and *λ* to maximize the likelihood using a standard M-step for CTMP (e.g., [57])

Note that, in Equation 4, we have used the simplification
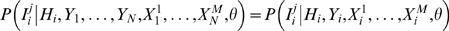
afforded by the conditional independencies in the generative model (Figure 3).

#### Computational requirements

In this section, we briefly outline the theoretical and practical computational requirements of the models. The running times are primarily a function of the number of target attributes |**Y**|, the number of predictor attributes |**X**|, and the number of individuals in the study *N*. |**Y**| and |**X**| slowly increase with *N*, as larger cohorts will have more observed HLA alleles and amino acids at each codon. In the present study, the HOMER cohort had values *N* = 567, |**Y**| = 1177, and |**X**| = 1242, and the combined HOMER-Durban cohort had values *N* = 1144, |**Y**| = 1287, and |**X**| = 1357. Roughly, analyses using these models scale as *O*(|**X**||**Y**| *N*log_2_ *N*), as we run one test for each *X*−*Y* pair and likelihood calculations on a tree are *O*(*N* log_2_ *N*). The number of EM iterations required to converge is roughly independent of the size of the data. In the case of the multivariate models, there is an additional penalty due to the forward selection procedure that requires a complete pass through all predictors to identify the most significant predictor for each iteration. Likelihood maximization is slower for Noisy Add, due to the increased number of iterations required for EM to converge for large numbers of significant predictor attributes, and inference being quadratic in the number of significant predictors.

In practice, all of the models were run on a 320 node Windows HPC cluster and completed in 1–24 hours, with the shortest times corresponding to the univariate model run on the smaller HOMER cohort and the longest times corresponding to the Noisy Add model run on the combined HOMER-Durban cohort.

## Results

### Model Validation on Synthetic Data

In this section, we use synthetic data to demonstrate the power and calibration of the proposed models and to demonstrate that failure to account for the phylogenetic tree, linkage disequilibrium (LD) among HLA alleles, *and* covariation among the amino acids will lead to a significant drop in power and inflation in estimates of significance.

#### Noisy Add represents real data better than Decision Tree

**Figure 7:**
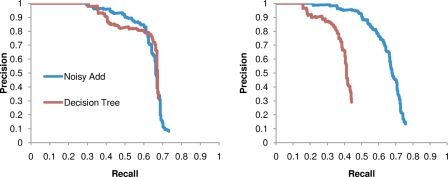
Noisy Add represents real data better than Decision Tree. Synthetic data were generated according to the Decision Tree model fit to real data (A) and the Noisy Add model fit to real data (B). On both datasets, the Noisy Add model performs at least as well as the Decision Tree model. In contrast, the Decision Tree model does poorly when applied to data generated from the Noisy Add model.

We have described two models that can each simultaneously account for the shared evolutionary history among viral sequences, linkage disequilibrium among HLA alleles, and covariation among the HIV amino acids. Before proceeding, it is useful to determine which of the two models better represents the real data. To examine this issue, we generated synthetic data from the HOMER Gag data according to (1) the Decision Tree model fit to real data (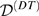), and (2) the Noisy Add model fit to real data (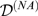). We then applied both models to both datasets. In general, the model that generated the data should be the optimal model for performing inference on that data. We indeed found this to be true in our experiments, but in addition, we found that the performance of the Noisy Add model was equivalent to that of the Decision Tree model on 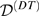 (there was no detectible difference between the PR curves; p = 0.46), whereas the performance of the Noisy Add model on 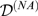 was significantly better than that of the Decision Tree model (*p*<0.0001) (Figure 7). Thus, the Noisy Add model appears to be better able to capture the relationships in the true data than the Decision Tree model. Consequently, in what follows, we concentrate exclusively on the Noisy Add model. We note, however, that the Decision Tree model is computationally more favorable and may be useful when resources are limited.

#### Covariation confounds simple tests

As we have discussed, there are at least three major sources of statistical confounding for HIV-HLA association tests: phylogeny (*P*), linkage disequilibrium among HLA alleles (*L*), and covariation among HIV codons (*C*). Previous approaches to finding HLA-associated polymorphisms have accounted for LD but not phylogeny [6], accounted for phylogeny but not LD [7], or accounted for phylogeny and LD but not covariation [8]–[11]. None of the previous approaches considered HIV codon covariation. To compare the relative contribution of each of these sources of confounding, we constructed five models that each account for a subset of the confounding sources as well as a baseline model that does not account for any source of confounding:

1. No correction for confounding (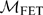). We use Fisher's exact test to compute exact *p*-values for associations between *X* and *Y* assuming *X* and *Y* are independent and identically distributed across individuals.
2. HLA LD only (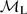). We use the Noisy Add model where only HLA-allele attributes are predictors and no correction for phylogenetic structure is made (achieved by fixing *λ* to be infinity). This model is similar to the one used by Moore et al. [6], except that Moore et al. used logistic regression rather than Noisy Add.
3. HLA LD and covariation only (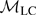). We use the Noisy Add model (where both HLA-allele attributes and attributes representing other codons are predictors) with no correction for phylogenetic structure (*λ* set to infinity). This model is similar to a second model in Moore et al., who suggested adding other codons as covariates to their logistic regression model [6]. Bhattacharya et al. later suggested that this approach could implicitly correct for some of the effects of the phylogeny [7]. As we shall see, it does when considering HLA-codon associations, but does not when considering codon-codon associations.
4. Phylogeny only (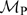). We use the univariate model where only HLA-allele attributes are predictors. This model is the second method described in [7] and fully evaluated in [21].
5. Phylogeny and HLA LD (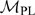). We use the Noisy Add model where only HLA-allele attributes are predictors. Matthews et al. [11] used this approach with the Decision Tree leaf distribution. Also, this model is similar to the approach described in [8],[10], wherein the univariate model in [7] is followed by an ad hoc post processing step that identifies HLAs in LD that are most likely to be responsible for immune pressure.
6. HLA LD, covariation, and phylogeny (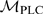). We use the Noisy Add model.

**Figure 8:**
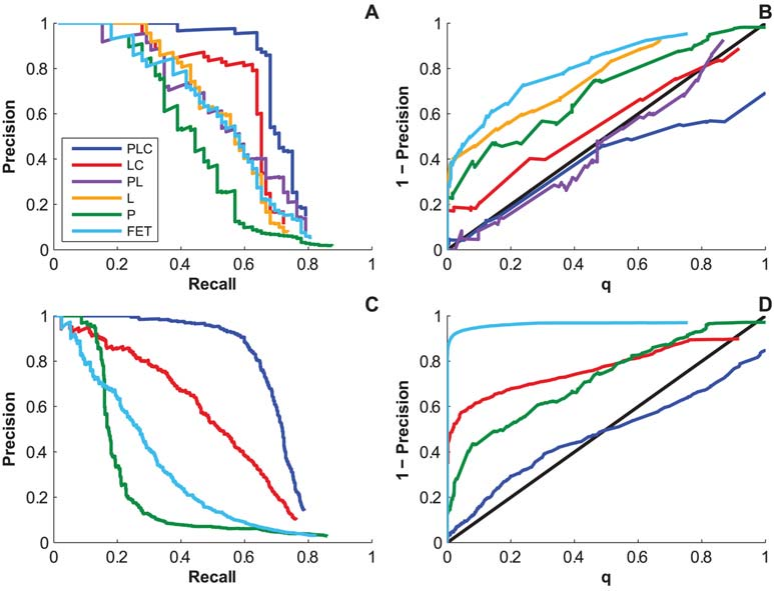
Performance on data generated from the 97% clade B HOMER cohort. Precision-recall (A) and calibration curves (B) of the models with respect to HLA-codon associations; precision-recall (C) and calibration curves (D) of the models with respect to both HLA-codon and codon-codon associations. Better precision-recall curves are ones that tend toward the upper right of the plot. Curves with perfect calibration follow the diagonal.

***Ability to identify direct HLA-codon associations.*** Because the primary purpose of previous studies has been to find HLA-mediated adaptations in the HIV genome, we first looked at the ability of these models to recover HLA-codon associations, ignoring codon-codon associations. Figure 8A shows the precision-recall (PR) curves for the six methods when run on synthetic data from the HOMER cohort. These curves indicate that all three sources of confounding play a significant role, and failure to account for any one of them leads to a dramatic drop in power. Although confounding due to both phylogeny and HLA linkage disequilibrium have been previously recognized [6]–[8],[10],[11], these curves demonstrate the significant confounding effect of codon covariation. As we have discussed, this observation can be explained by the failure of the univariate model to distinguish between direct and one-hop associations. Although both associations may be considered HLA associations, there are practical implications to distinguishing a direct association, which is likely to be the primary (e.g., most common, rapidly selected or necessary) escape mutation in an HLA-restricted epitope, and an indirect association, which may (e.g.) compensate for fitness costs introduced the primary escape mutation or provide further escape in the context of the primary escape mutation.

It is interesting to note that accounting for phylogeny and linkage disequilibrium (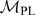) does not appear to increase power over accounting for linkage disequilibrium alone (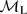) or even baseline (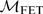), and accounting for all three confounders (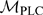) has only a modest (but significant, *p* = 0.009) increase in power over accounting only for linkage disequilibrium and codon covariation (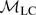). One reason may be the relative homogeneity of the HOMER cohort (97% clade B), which limits the amount of power that can be gleaned from the phylogeny. It is important to note, however, that *any* unaccounted for structure in the data will lead to an increased bias in the LRT and thus the *q* statistic [21]. This effect is seen here in the poor *q*-value calibration of the phylogeny-naïve models shown in Figure 8B. Only the models that account for at least phylogeny and LD (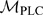 and 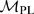) have calibrated *q*-values. In contrast, the models that do not account for phylogeny or linkage disequilibrium grossly exaggerate significance.

***Ability to identify codon covariation.*** The fact that codon covariation significantly confounds HLA-codon association statistics suggests that many of the codons are strongly influenced by polymorphisms at other positions. Indeed, prediction of covarying amino acids has a rich literature, with most methods unable to scale beyond pairs of covarying amino acids or to statistically account for the shared phylogeny [22],[37]. We therefore measured the ability of 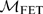, 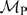, 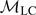 and 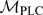 to recover codon-codon associations in addition to HLA-codon associations.

The full Noisy Add model (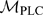) achieves roughly the same power as it did for HLA-codon associations (≈70% recall at 20% *q*-value; Figure 8C). In contrast, failure to account for phylogenetic confounding (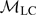) significantly reduced power (*p*<0.0001), despite the relative homogeneity of the data. Furthermore, accounting only for phylogeny (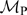), as many codon-covariation models have proposed [12], [20], [22]–[31], performed even worse, reflecting a tendency to pick up indirect associations. At high precision (>70%), accounting for only phylogeny improved performance relative to baseline, though at lower precision Fisher's exact test outperformed the more error-prone LRT-based 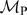. In addition, the phylogeny-only (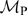) and the phylogeny-naïve (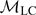, and 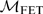) models were extremely poorly calibrated (Figure 8D), indicating that *q*-values produced by these models are misleading. In the following section, where we consider multi-clade data, we shall see a more dramatic example of this failure. Thus, these experiments demonstrate the importance of accounting for both phylogeny and multivariate covariation when inferring correlated evolution among codons, even in relatively homogeneous cohorts.

#### Results on multi-clade synthetic data

**Figure 9:**
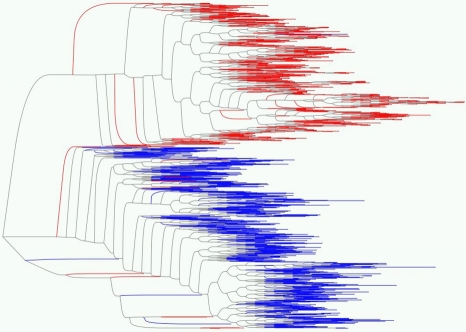
Tree built from the combined HOMER (red) and Durban (blue) cohorts [109]. In the text, “clade B” refers to the predominately red subtree and “clade C” refers to the predominantly blue subtree.

Comparing recent large cohort studies [8]–[11] to previous smaller studies [6],[7],[21] suggests that more associations can be detected by increasing sample size, a result directly confirmed by Rousseau et al. [10]. Although substantially increasing the size of existing cohorts may not be feasible, existing cohorts can potentially be merged together. One problem with this approach is that different cohorts typically consist of populations sampled from different geographical areas that differ substantially in HIV subtype distributions and ethnic composition (and thus HLA allele distribution), and the traditional approach of stratifying by clade and/or demographics defeats the purpose of increasing sample size. By correcting for the phylogenetic structure of the sequences, however, we can attempt to exploit the larger sample size of the combined data. At the same time, we can examine the similarities and differences among associations in different clades. To do so, we combined the HOMER and Durban cohorts, yielding a mixed-clade group of 1144 individuals, with a roughly equal mix of clades B and C (Figure 9).

**Figure 10:**
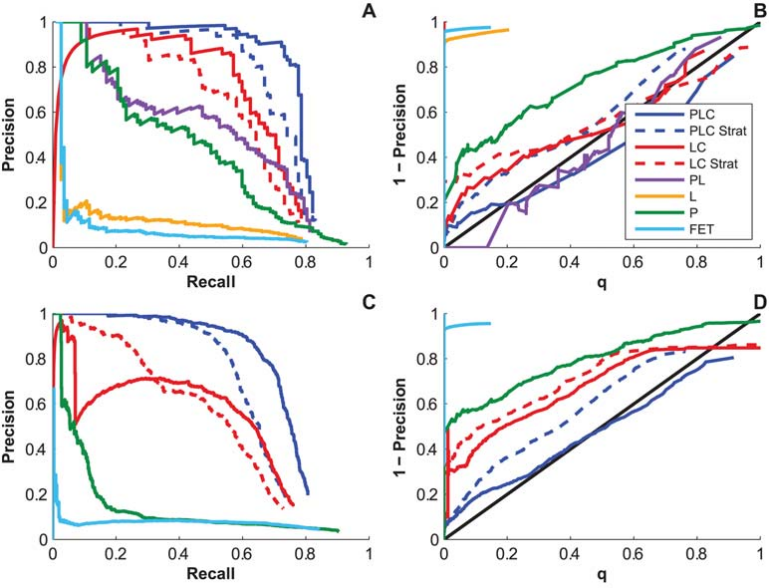
Performance on data generated from the mixed-clade B/C dataset. Precision-recall (A) and calibration curves (B) of the models with respect to HLA-codon associations; precision-recall (C) and calibration curves (D) of the models with respect to both HLA-codon and codon-codon associations. “PLC Strat” and “LC Strat” refer to running 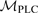 and 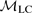, respectively, on data stratified by clade. The curves reflect the combined results from the two strata.

As in the previous experiments, we fit the full Noisy Add model to this combined dataset and then generated synthetic data from the resulting model. We then attempted to learn back the associations using the full dataset and then, for comparison, by stratifying the data and running the Noisy Add model separately for each clade. As indicated by the PR and calibration curves (Figure 10), the Noisy Add model successfully accounted for the heterogeneity in the data, as it remained calibrated and successfully recovered 80% of HLA-codon associations and 75% of all associations at 20% FDR. Importantly, the model demonstrated higher power on the combined dataset than on the stratified data (*p*<0.0005 for both HLA-codon associations only and all associations), indicating that there is shared information at both the HLA-codon and codon-codon levels and that power can be increased by merging datasets from disparate cohorts as long as all three sources of confounding are accounted for.

We then applied the remaining five models to this mixed-clade dataset, observing the founder effects demonstrated by Bhattacharya et al. [7]. In particular, using either Fisher's exact test (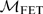) or accounting for LD alone (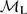), as proposed by Moore et al. [6], results in strikingly poor PR curves (Figure 10A) with calibration plots that indicate it is impossible to achieve greater than 10% precision (Figure 10B). These results are due to the founder effects demonstrated in [7] that arise from the fact that both HLA allele and HIV clade frequencies differ between human populations in different geographical areas. In contrast, using phylogeny alone (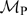) to account for founder effects, as proposed by Bhattacharya et al. [7], greatly increases power in the PR curve, though calibration is still poor. In this case, accounting for LD in addition to phylogeny (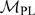), as proposed by Brumme et al. [8], only moderately increases power, though it corrects the problem with calibration. Similar to the results for single clade data, accounting for LD and codon covariation (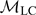) yielded further improvements in both power and calibration, though we note the peculiar nature of the PR curve, which indicates that the most significant associations are spurious. This peculiarity is even more pronounced when looking at both HLA-codon and codon-codon associations (Figure 10C). Inspection of the strongest spurious associations indicates that they are founder effects that serve as clade markers—meaning the strongest associations simply identify a sequence as clade B or clade C. Once these markers are accounted for in the model, the performance of the model begins to improve (where the right-hand-side of the curve increases with recall). In contrast, failure to account for any confounding (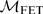) results in a strikingly poor PR curve.

Given the prominent structure of the multiclade data, a natural solution is to stratify the data by clade, running the phylogeny-naïve model (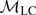) separately on each clade. Although stratifying the data removes the strongest founder effects, the overall performance is not significantly different from 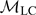 without stratification (*p* = 0.34 for HLA-codon associations and *p* = 0.09 for all associations). Nevertheless, it is interesting to note that, in the HLA-codon case, after the founder effects are incorporated into the model, the non-stratified version of 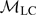 appears to perform better than the stratified version, reinforcing the observation that there are common sites of escape in the two cohorts. Unfortunately, it is impossible to distinguish founder effects from true signal, limiting the practical value of this approach.

In contrast, accounting for phylogeny with the full model (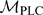) significantly outperformed both the stratified and non-stratified versions of the phylogeny-naïve model (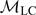) on both types of associations (*p*<0.0001 in both cases) and did not suffer from founder effects. Finally, it is striking that for codon coevolution, it is better to account only for protein-wide codon covariation than to use a sophisticated phylogenetic-correction algorithm that is limited to pairwise associations, especially if the data can be stratified by gross tree topology (in this case, HIV clades), although accounting for both phylogeny and codon covariation is clearly a more powerful approach.

#### Noisy Add can distinguish among specific leaf distributions

As discussed in Methods, the Noisy Add and univariate models incorporate a model of selection pressure for each predictor attribute that can take one of four forms: escape, reversion, attraction or repulsion. Furthermore, although escape and reversion (attraction and repulsion) are negative (positive) correlations, each process is distinct. So far, we have assumed that the primary purpose of these studies is to uncover associations at a codon level, and so ignored the specific leaf distribution learned. Nevertheless, the leaf distribution may be informative. To determine how well the model can recover the true leaf distribution, we compared the leaf distribution of the model instance used to generate the synthetic data with that learned from the synthetic data. On the three synthetic datasets we have discussed (synthetic data generated by Decision Tree and Noisy Add leaf distributions on the HOMER cohort, and synthetic data generated with Noisy Add leaf distributions on the combined HOMER and Durban cohorts), the Noisy Add model recovered the correct leaf distribution in 85–90% of cases where it recovered the correct predictor-target pair. It should be noted, however, that the forward selection scheme used by Noisy Add makes it unlikely that both complementary processes (escape/reversion or attraction/repulsion) will be recovered, even if both processes are present. Thus, when we find (e.g.) an escape association, it does not preclude the presence of a reversion association.

There is great utility in being able to distinguish between escape and reversion processes. Escape is an indication of CTL pressure, whereas reversion in the absence of the allele is as indication of replicative cost to the escape variant that leads to active reversion (as opposed to passive drift) in the absence of CTL-mediated selection pressure. Consistent with these interpretations, Matthews et al. [11] recently showed that reversion associations but not escape associations correlate with reduced plasma viral load in chronic infection. More generally, of course, the biological interpretation of these distribution forms will vary across domains of application.

#### Statistical power as a function of sample and effect sizes

Previous studies have differed widely in their results, in part because they employed different methods, and in part because they used different sample sizes (473 [6], 96 [7], ≈550 [8], 181 [21], 261–452 [10], and 262–666 [11]), which affects the power to detect associations. Not surprisingly, the larger studies found more associations, which suggests even larger studies may be beneficial. It is important to note, however, that the adverse effects of violating model assumptions increases with sample size, as assumption violations lead to deviation from (and statistical rejection of) the null distribution (see, for example, [58]).

**Figure 11:**
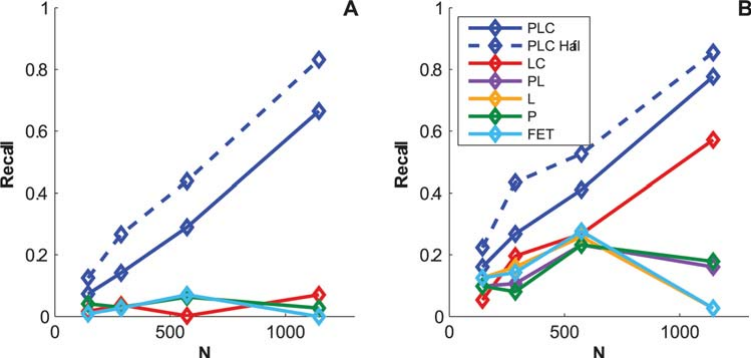
Power to detect both HLA-codon and codon-codon associations (A) or just HLA-codon associations (B) in the mixed-clade cohort at 80% precision. The “PLC Half” curve plots the power of 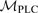 on synthetic data generated using only associations that were identified from a cohort one half the size of the full cohort. The curves show how power is affected by the strengths of the planted associations.

To measure the effects of sample size on the power of the six methods in consideration, we created additional synthetic datasets of size 143, 286, and 572 by randomly selecting 12.5%, 25% and 50% of the individuals from the mixed-clade synthetic dataset. Figure 11 shows the dramatic increase in power that the full Noisy Add model (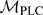) experiences as a function of *N*. Here, power is defined to be the ability to detect associations at 80% precision regardless of the model's reported *q*-value. In the range tested, the Noisy Add model has an approximately linear increase in the power to detect associations (HLA-codon and codon-codon associations combined) as the sample size increases. In contrast, increasing sample size for the other models has a limited effect. In particular, failure to account for codon covariation leads to a flat power curve for all models that do not account for codon covariation. The model that accounts for codon covariation and HLA allele LD but not phylogeny (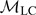) does experience a linear increase in power to detect HLA-codon associations (but not codon-codon associations), though the power is less than that of the phylogenetically-corrected model (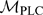) at all sample sizes. Only the full model (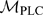) experiences any increase in power to detect codon-codon associations. Thus, simply increasing the cohort size will not lead to an increase in power if improper models are used. Rather, model calibration is likely to be negatively impacted as large numbers of spurious (yet non-null) associations are detected. Similar trends were seen on the HOMER (clade B only) cohort (data not shown).

Statistical power is a function of sample size and effect size. In the results just described, the planted associations came from those detected at 20% *q*-value on real data at *N* = 1144. If we instead plant associations detected at 20% on real data at (e.g.) *N* = 572, those associations will presumably be stronger and hence the measured power should be greater. To demonstrate this supposition, we ran Noisy Add on a random subsample of the mixed clade cohort, and then generated new synthetic datasets based on these associations. The dashed blue lines (labeled “PLC Half”) in Figure 11A and 11B show the increased power to detect planted associations originally found at *N* = 572 compared to planted associations originally found at *N* = 1144. Thus, if associations of the strength detected by Brumme et al. [8] (*N*≈550) are desired, then *N* = 1150 will provide sufficient power to recover 90% of the associations. If, however, more subtle effects are sought, then larger cohorts are necessary. It is our opinion that only post hoc analyses of larger cohorts will determine the minimum relevant effect size. Furthermore, it should be noted that, whereas power can be increased by combining data from multiple cohorts, if only associations for a single cohort are of interest, then greater power will clearly be achieved from a single-clade cohort of size *N* than from a multi-clade cohort of size *N*. Nevertheless, the power increase from combining cohorts of different clades will prove useful in situations where single-clade cohorts cannot be expanded in practice.

### Phylogenetic Dependency Network for Gag p17 and p24

**Figure 12:**
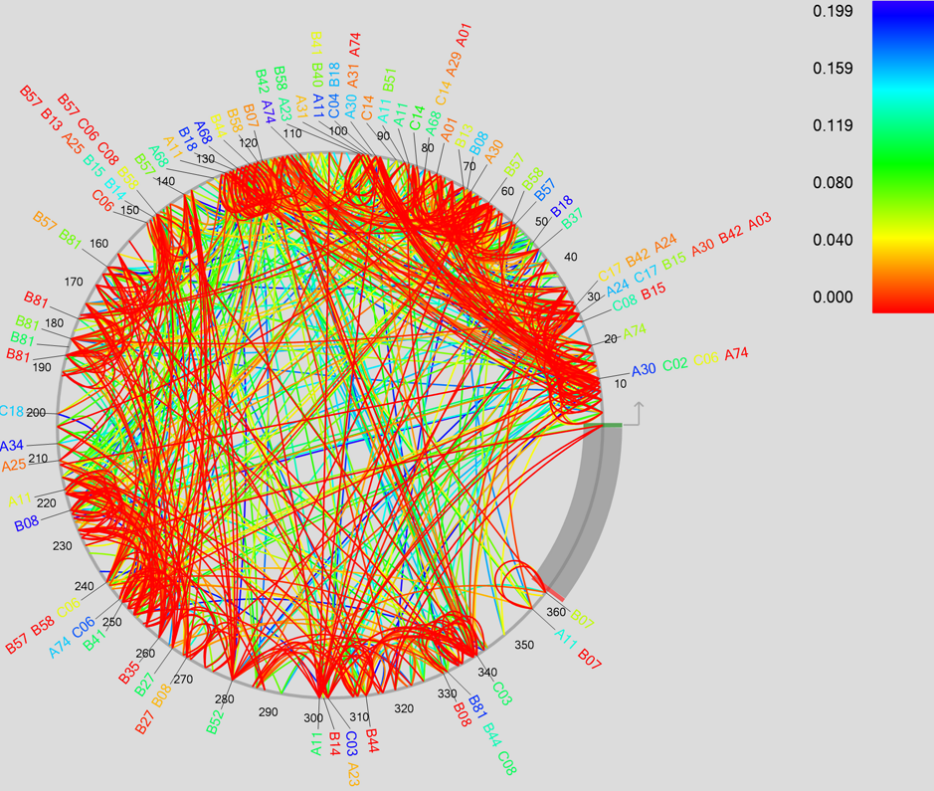
Gag phylogenetic dependency network for combined HOMER and Contract cohorts. Gag p17 and p24 are drawn counterclockwise, with the N-terminus of p17 at the 3 o'clock position. Arcs indicate association between codons (inside the circle) or between HLA alleles and codons (outside the circle). Colors indicate *q*-values of the most significant association between the two attributes.

Having established the Noisy Add model's ability to reliably recover associations in mixed clade datasets, we now turn to an analysis of the actual associations that were recovered on the mixed clade B/C data. The Noisy Add model found 149 HLA-codon associations and 1386 codon-codon associations at *q*≤0.2, representing 100 distinct HLA-codon pairs and 716 distinct codon-codon pairs. To explore these dense networks we developed a dependency-network viewer, PhyloDv, designed for intra-protein networks. PhyloDv draws the protein as a circle, with the N-terminus at the “3 o'clock” position and the protein extending counter-clockwise around the circle. Codon-codon associations are drawn as headless arcs (or edges) within the circle, whereas HLA-codon associations are drawn as external edges. Edge color reflects the strength of the association. Figure 12 shows the full Gag dependency network at 20% *q*-value. The program, which includes interactive detailed views of each codon to explore the specific associations, is available upon request. The individual associations are available as Table S1.

The dense PDN reveals broad patterns of codon covariation and HLA-mediated substitutions. For example, pairs of codons are more likely to covary within a subprotein (N = 528) than between subproteins (N = 188; *p*<10^−31^), and a disproportionate number of p17 codons (24%) are associated with HLA alleles than are p24 codons (13%; *p* = 0.009). Not surprisingly, a disproportionate number of covarying codons were within 10 positions of each other (162/716; *p*<10^−55^). (We compute *p*-values by using Fisher's exact test to estimate the significance of a contingency table that compares observed associations against *null codon pairs*, which we define to be the set of all codon pairs that were not called significant by the PDN but which did pass the minimum count pre-processing filter.)

Interestingly, of the 62 codons that are associated with at least one HLA allele, 59 (95%) predict substitutions at other codons. Furthermore, on average, each HLA-associated codon predicts substitutions at 7.0 other codons on average (range 0–25). These two observations highlight the complexity of HLA-mediated escape. Also of note is that, of the 181 codons that covary with at least one other codon, 60 (33%) have an association with at least one HLA allele, suggesting that HLA-mediated selection pressure will confound codon coevolution when unaccounted for.

Among the 68 HLA-codon associations with escape/reversion leaf distributions, 33 represent escape/reversion from a residue that is consensus in both clades B and C, 7 represent escape/reversion from clade B consensus only, and 11 represent escape from consensus C only, where we define clade B consensus based on the HOMER cohort and clade C consensus based on the Durban cohort. Interestingly, of the 11 clade C susceptible associations, 5 had predicted resistant forms matching clade B consensus (A*29 F79Y, A*68 F79Y, B*35 D260E, A*11 F301Y, B*44 D312E), and 2 of the 3 clade B susceptible associations had a predicted resistant form matching clade C consensus (A*01 Y79F, A*31 R91K). In all, there were 21 HLA-codon associations for which the predicted resistant form was clade B or C consensus (Table 1). These associations may represent instances in support of the “HLA footprinting” hypothesis, which states that the current circulating viral sequences are a reflection of escape from prominent HLA alleles in different human subpopulations [6],[59]. Indeed, 17 of these 21 associations involved common HLA alleles that are found in at least 10% of individuals in at least one of the two cohorts (Table 1). Four of these 21 associations lie in optimal epitopes, which is reasonable given that such responses are less likely to be identified using overlapping peptide scanning technologies that seek to maximize consensus sequence coverage. In three of those four optimal epitopes, the predicted susceptible form matches the optimal epitope. B*07-associated S357G is the one exception, where G is both clade B and C consensus and is also the amino acid in the optimal epitope sequence. This association may represent an instance where the so-called optimal epitope was actually a partially escaped form. It is interesting to note however that B*07 is a very common allele in both the HOMER and Durban cohorts and, in one recent study, all studied optimal B*07 epitopes in Gag, Pol and Nef were found to contain at least one association predicting that the optimal epitope actually contained an escape polymorphism [60].

**Table 1:** HLA-codon associations in which clade B and/or clade C is the predicted resistant form.

				Consensus		HLA Freq (%)		
HLA	Pos	Susc	Res	B	C	B	C	Optimal
A*01	76	R	K	K	K	23.5	9.9	
A*01	79	Y	F	Y	F	23.5	9.9	
A*11	93	X	E	E	E	12.3	0.3	
A*11	301	F	Y	Y	F	12.3	0.3	
A*24	**30**	**K**	**R**	**R**	**M**	**19.2**	**5.4**	**KYKLKHIVW**
A*29	79	F	Y	Y	F	8.4	15.5	
A*30	67	X	A	S	A	5.2	34.1	
A*31	91	R	K	R	K	8.0	0.9	
A*68	79	F	Y	Y	F	9.3	24.2	
A*74	109	X	N	N	N	0.3	9.9	
B*07	**357**	**S**	**G**	**G**	**G**	**23.1**	**9.9**	**GPGHKARVL**
B*14	147	X	I	I	I	6.5	5.5	
B*15	28	X	K	K	H	18.8	34.8	
B*15	147	X	I	I	I	18.8	34.8	
B*35	**260**	**D**	**E**	**E**	**D**	**16.9**	**3.6**	**PPIPVGDIY/NPVPVGNIY**
B*42	30	X	R	R	M	0.4	22.4	
B*44	**312**	**D**	**E**	**E**	**D**	**19.5**	**14.9**	**AEQASQDVKNW**
B*57	54	A	S	S	S	6.4	9.5	
B*81	163	X	A	A	A	0.1	11.8	
C*06	146	P	A	A	A	13.7	28.3	
C*06	242	N	T	T	T	13.7	28.3	

Bold lines match optimal epitopes. X indicates no significant association.

As noted in the synthetic results, Noisy Add can distinguish with 85% accuracy the difference between reversion and escape leaf distributions (though it cannot discern whether both are present). On the current dataset, 5 HLA-codon associations were identified as primarily reversion: B*14 K302, B*15 K26, B*57 T242, B*58 G55, and B*81 L184. These associations most likely have a corresponding escape association that we are not detecting, but these associations are nonetheless notable in that there is a strong statistical pull towards the “susceptible” form in the absence of the associated allele, which may suggest that fitness costs are associated with the resistant form [4],[11],[46]. Indeed, in the case of T242, the resistant form N242 is known to reduce *in vitro* fitness [39],[46].

#### Known escape pathways are predicted by the PDN

In some cases, CTL escape requires a set of secondary substitutions that may stabilize protein structure, compensate for lost protein function, or facilitate further escape [38]–[40], [61]–[66]. To date, however, the identification of such complex pathways has been largely limited to studies of single immunodominant epitopes restricted by HLA alleles that are known to be protective against infection. The PDN systematically predicts potential escape pathways across all epitopes and HLA restrictions. Here, we assess the quality of these predictions by checking to see whether well-studied escape pathways are found in our PDN.

The first HIV escape pathway that was described in detail is escape from the B*27-restricted KK10 epitope in p24 Gag 263–272 [61],[67]. In the mid 1990s, it was demonstrated that the R264K/G mutations abrogated B*27 recognition of the KK10 epitope [67],[68]. Here, we find that B*27 is strongly correlated with escape from R264 (Noisy Add *q* = 0.01), with the result being evenly distributed between K (*q* = 0.08) and G (*q* = 0.11). Kelleher et al. later reported that the R264K but not R264G mutation was typically preceded by L268M. Accordingly, in the PDN, we find that L268M predicts R264K (*q*<0.001) but not R264G, and that L268M is itself predicted by B*27 (*q* = 0.001). Kelleher et al. also reported that the R264G was associated with E260D, and Schneidewind et al. [41] confirmed that E260D compensates for R264G but not for R264K. We find the same association in the PDN. (Note that, although E is clade B consensus and D is clade C consensus, every individual in both cohorts with G264 has D260).

Recently, Schneidewind et al. demonstrated that S173A compensates for the loss of replicative capacity incurred by R264K [38]; this R264K substitution (but not R264G) is strongly associated with S173A in our model (*q*<0.001). In addition, we note that R264K is strongly associated with substitution I267V (*q*<0.001) within the KK10 epitope and with L215M (*q* = 0.01). Residue 264 is within 3 angstroms of both codons 215 and 173 in folded p24 [69], which may explain the compensatory relationship between codons 173 and 264 [38] and predicts a similar relationship between codons 264 and 215.

Finally, although it is not known if there are any determinants that predict whether KK10 escape occurs via the R264K or R264G pathway [41], we find several associations that predict one pathway or the other. Most strikingly, of the 7 individuals with R264G, 4 have Q136R (*q* = 0.0001), a substitution which also strongly predicts the D260E substitution of the R264G pathway (*q*<0.001). In addition, A146P is associated with maintaining wild type L268 (not the R264K pathway) whereas A163X predicts I267V (R264K pathway). Both A146P [52] and A163X [40] are B*57-mediated escape substitutions (see below), though no individuals in the cohorts are both B*57 and B*27 positive, making interpretation difficult.

The B*57 and B*5801 alleles have been strongly associated with effective HIV control [70]–[73], an effect that may be due in part to successful targeting of Gag epitopes [44] and the high cost to viral load of CTL escape from some epitopes targeted by these alleles [39],[74]. Recently, the details of escape from the B*57-restricted TW10 epitope in Gag codons 240–249 have been described [3],[39],[74]. TW10 escape begins with a T242N escape mutation, which partially abrogates B*57 binding, but also elicits a measurable fitness cost to the virus in part by disrupting cyclophilin A (cypA) interactions [74]. The fitness costs of this mutation may be partially restored by compensatory substitutions H219Q, I223V and M228I [39]. This escape pattern is captured by the dependency network, which finds a direct HLA-codon association between B*57 and 242 (*q*<0.001). The T242N substitution predicts (*q*<0.001) further escape at G248A (position 9 of the TW10 epitope) and a single compensatory mutation at codon E210D (*q* = 0.01) in the CypA binding loop, whereas the G248A substitution predicts compensatory substitutions V218A (*q* = 0.02) and M228V (*q* = 0.08), and G248T predicts H219Q (*q* = 0.07) and M228I (*q*<0.001), of the CypA binding loop. Although 228 is (in at least some structural models) in direct contact with 248 (3 angstroms) [69], the other associations are more distant (10–20 angstroms). Nevertheless, the CypA substitutions have been shown to compensate for the 242 and 248 mutations [39], underscoring the fact that compensatory mutations may be of a more functional nature and not strictly due to protein structural constraints.

Previous studies have reported alternative escape pathways in the A*02-restricted SLYNTVATL epitope (Gag positions 77–85) at epitope positions 3, 6, and 8 (though the Y79F escape at epitope position 3 is clade C consensus) [65]. Although the model finds associations with three HLA alleles that restrict known epitopes that overlap this region (A*01, A*11, A*29), no correlations were observed with A*02. The lack of signal may be due to several factors that will each dilute the signal: multiple escape pathways that occur in different sites, dilution from the overlapping epitopes for which there is a stronger signal, and evidence that a lack of fitness cost will lead to low rates of reversion [65], which, coupled with the high rate of A*02 in the population (40.5% in the combined cohort), suggests that many non-A*02 individuals will have escape variants.

#### Codon covariation reflects three-dimensional protein structure

In the first study of its kind, Poon and Chao [35] reported that 70% of artificially induced, fitness-reducing mutations selected for partially restoring compensatory mutations in the DNA Bacteriophage *φ*X174. No studies have systematically explored this phenomenon for immune escape in HIV Gag or other viruses, but the case studies of B*27 KK10 and B*57 TW10 make it clear that compensation happens in response to at least some CTL escape mutations. Poon and Chao further reported that compensatory mutations tended to cluster in linear and/or three-dimensional space, though many exceptions were noted. Indeed, the KK10 and TW10 case studies reveal two patterns of compensation: *distal compensation* in the case of TW10, where compensatory mutations are distal in three-dimensional space but alter functional dependencies, and *proximal compensation* in the case of KK10, where codon pairs in a compensatory pathway are in close proximity and are likely required to maintain structural fidelity. Although the PDN predicted both known pathways, only the latter form of compensation can be easily and independently verified computationally by computing distances between covarying codon pairs.

To determine the proportion of covarying codon pairs that are in direct contact, we computed codon-codon distances against the p17 trimer crystal structure [75] and the p24 and p17p24 polyprotein NMR structures [69]. The distances were computed as the minimum distance between any reported atoms for each codon in a single PDB model, taken over all models and all three structures. The distances for the p17 trimer crystal structure tended to be farther than for the p24 and p17p24 NMR structures as hydrogen atoms, which tend to be on the periphery of amino acid molecules, are not mapped in crystal structures. To compute the significance of the results, we also computed three-dimensional distances among *null codon pairs*, which we defined to be all codon pairs for which no significant direct associations were found even though both codons exhibited enough variability to pass our minimum count filter.

Among the 424 significant (*q*≤0.2) *linearly distal codon pairs* (>10 codons apart) that could be mapped to at least one structure, 37 (8.7%; *p*<10^−11^, Fisher's exact test against null codon pairs) were within 5 Å of each other and 113 (26.7%; *p*<10^−20^) were within 10 Å. Even among *linearly proximal* codon pairs (2–10 codons apart), covarying pairs were more likely (75/121; 62%) than non-covarying pairs (1075/2417; 44%) to be within 5 Å in the three-dimensional structure (*p* = 0.0001).

To further validate the ability of the model to distinguish direct associations within a chain of interactions, we computed pairwise distances among all linearly distal one-hop associations, excluding instances where a direct association was also inferred. The median distance between direct association codon pairs (15.9 Å) was significantly smaller than the median distance between one-hop codon pairs (19.2 Å, *p*<0.0001). The direct codon-codon associations were also significantly closer than those of 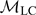 (median 18.4 Å, *p* = 0.003; 5.3%<5 Å, *p* = 0.04), which doesn't account for phylogeny, and 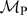 (median 21.8 Å, *p*<10^−12^; 3.2%<5 Å, *p*<10^−5^), which computes pairwise, phylogenetically-corrected associations. Fisher's exact test (median 22.1 Å) was indistinguishable from the null codon pairs (median 22.9 Å, *p* = 0.63).

The complete set of distances is reported as Table S2. It should be noted that long range distances do not preclude a compensatory relationship, as long range effects are common [35] and both p17 and p24 form complexes, suggesting that some structural compensations may exist at the interface between two instances of the same protein. Nevertheless, those codon pairs for which we observe both strong dependencies and colocalization in the three-dimensional structure are strong candidates for further study with regards to compensation.

#### Codon covariation reflects correlated epitope targeting

The epitopes targeted by CTL are not a simple function of the individual's HLA repertoire. Rather, specific patterns of epitope targeting are often observed. For example, epitope targeting by CTL often follows patterns of immunodominance [76], wherein initially only one or a few epitopes (the *dominant* epitopes) are strongly targeted by the T-cell response. However, a shift in immunodominance patterns occurs over the course of infection, as the T-cell response broadens to target additional epitopes [77],[78]. Given that patterns of immunodominance appear to be largely consistent at the population level in at least some cases [3],[60],[79],[80], the sequential selection of escape mutations restricted by the same HLA allele that results from sequential targeting of HLA-restricted epitopes over the course of infection may also be reflected as patterns of codon covariation. In the case where escape is sequential, escape in subdominant epitopes may be better predicted by escape in dominant epitopes than by the presence of the restricting HLA allele. To use the immunodominant B*57 allele as an example, the earliest and most frequently targeted B*57-restricted epitope is TW10 [70]. TW10, however, is not the only B*57-restricted Gag epitope. Other epitopes exist in codons 162–172 (KF11) [40], 147–155 (IW9) [52], and 308–316 (QW9) [51]. Recent results indicate that TW10 tends to escape most rapidly, followed by IW9 then KF11 [60] (QW9 was not studied). On the combined Durban-HOMER dataset, the dependency network predicts direct HLA-codon associations between B*57 and codons in TW10, IW9, KF11 and positions 54–62, which we'll refer to as putative pSG9 epitope, as well as striking codon covariation. For example, the antigen-processing escape A146P [52] (one codon upstream of the IW9 epitope) is predicted by both the presence of B*57 *and* the presence of the T242N TW10 escape mutation, suggesting that escape in IW9 often occurs in the context of escape in TW10 (but not always, as indicated by the direct B*57-146 association). Similarly, A163G KF11 escape is predicted by escape substitutions T242N (TW10) and I147M (IW9), and lack of escape at 310 (QW9), reflecting previous reports of the targeting order of Gag B*57-restricted epitopes [40],[60],[70], whereas pSG8 escape is correlated with escape at TW10, IW9 and QW9.

It is important to note that the order in which direct escape associations arise cannot be inferred from the PDN. Rather the presence of arcs between epitopes suggests that targeting of epitopes restricted by the same allele is somehow correlated. Immunodominance is one biological mechanism that may induce such *CTL-mediated* codon covariation. Another may be the overall strength of the CTL response and/or the strength of the CTL response to epitopes targeted by a given allele. In the most extreme example, epitopes are either targeted or not depending on the strength of the immune system. Individuals who are targeting the epitopes will tend to select for escape mutations in all the epitopes, whereas individuals who have weakened immune systems may not target any of the epitopes (or will target with less strength). In this scenario, escape from epitope A implies that the immune system is active, thus increasing the likelihood of escape from epitope B, and vice versa. In a less extreme example, suppose that some individuals with a given allele mount a response to epitopes restricted by that allele, whereas other individuals do not. This situation will lead to codon-codon dependencies among associations in epitopes restricted by the allele. In addition, several studies have noted the accumulation of multiple or alternative escape substitutions within the same epitope [8],[10],[21],[38],[40],[41],[46],[65],[81],[82]. We would therefore expect to see codon-codon dependencies within the same epitope as well.

To determine how much of the observed codon covariation may be CTL-mediated, we looked at covariation in the PDN with regard to known, optimally defined epitopes. Among linearly proximal codon pairs, both co-evolving codons were within the same HLA-restricted optimal epitope 138 of 162 times (85.2%, compared to 72.0% of null codon pairs; *p* = 0.0002). 213 of 554 (38.4%; *p* = 0.003) linearly distal codons pairs occurred within different optimally-defined epitopes restricted by the same HLA allele (compared to 32.3% of null codon pairs). If we also include predicted epitopes, defined here as the region ±5 codons from a direct HLA-codon association, then 304 (54.8%; *p*<10^−17^) linearly distal codon pairs are in known or predicted epitopes restricted by the same HLA allele (compared to 36.6% of null codon pairs). We thus conclude that a majority of codon covariation in Gag p17/p24 is attributable to CTL-mediated selection pressures, though the specific mechanism of CTL-mediated covariation cannot be identified from this study.

#### Direct HLA-codon associations map to known epitopes

The observation that a majority of codon-codon associations occur within or proximal to epitopes restricted by the same HLA allele suggests that CTL escape is driving much of the observed HIV codon variation. Indeed, Brumme et al. [60] recently showed that at least 36% of observed Gag substitutions in acutely infected individuals are due to HLA-associated polymorphisms (possibly including indirect associations), a proportion that may increase once the full PDN is considered. It may therefore be surprising that there are only 100 direct HLA-codon associations. The synthetic studies showed that the Noisy Add model can successfully recover the primary escape mutations and is not prone to hallucinating indirect associations, indicating that we can assume the direct–indirect distinction with some confidence. Thus, there appears to be a dense network of correlated escape among epitopes, with a relatively sparse set of primary escape mutations that are most rapidly and/or most frequently selected for. Teasing apart the underlying causality and accuracy of this network requires a large number of longitudinal samples and laborious experimental data. Nevertheless, the accuracy of the PDN can be approximately tested by evaluating which associations lie in optimal epitopes. Specifically, if the PDN is accurate, then direct associations are more likely to lie in or near epitopes than are one-hop associations (*H*→*B* in the *H*→*A*→*B* chain, where *H*→*B* is not directly inferred by the algorithm). Thus, we categorized every direct and one-hop association based on whether or not it was within three codons of an optimally-defined epitope, using a strict matching criterion that required that an optimal epitope exactly matched the consensus sequence among either clade B or clade C HIV sequences that had the predicted susceptible amino acid and the codon in question.

**Figure 13:**
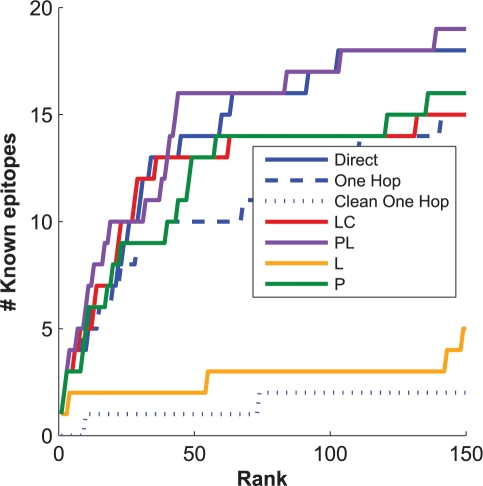
Number of associations in optimal epitopes as a function of *q*-value rank.

Figure 13 shows the number of in-epitope associations found as a function of the *q*-value rank of the association. To prevent double counting, only the most significant association per HLA-epitope pair was considered (see Methods). The plots suggest that direct associations may be more likely than one-hop associations to lie in epitopes, although the difference is not statistically significant. Given that most codon-codon covariations are between epitopes restricted by the same allele, it should not be surprising that many one-hop associations lie in epitopes. We thus additionally plotted only those one-hop associations that did not lie in an optimal epitope that was already predicted by a direct association (Figure 13, *clean one-hop*). Only four such associations were found with *q*≤0.4 (*p*<0.0001 compared to direct associations with a permutation test), indicating that most of the one-hop epitopes were epitopes that additionally had direct associations. It is therefore not surprising that 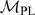, which fails to account for codon-codon covariation, identified escape mutations within almost as many optimal epitopes as the full model 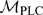 (Figure 13).

We further compared the HLA-codon associations of the other three models to the optimal epitopes. Only 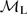 and 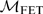, which fail to account for both phylogeny and codon covariation and are thus quite prone to founder effects [7], performed significantly worse than the other models (*p*<0.0001). The models that roughly account for clade differences, either through codon covariation (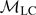) or phylogeny (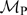), performed slightly worse than the full model, though these differences were not significant.

## Discussion

This study presents the first approach to simultaneously account for viral phylogeny, codon covariation, and HLA linkage disequilibrium in population-based association studies. It is also the first large scale multiclade analysis of HLA-mediated escape in HIV-1, as well as the first approach that simultaneously accounts for HLA linkage disequilibrium, HIV ancestral relationships, and codon covariation. The large number of direct HLA-codon associations confirms a substantial role of the HLA-restricted CTL response in driving HIV evolution, and supports the observation that patterns of HIV evolution are broadly predictable based on host immunogenetic profiles [6]–[10],[83]. Moreover, results demonstrate that escape and reversion mutations often arise in the context of a complex set of correlated substitutions that reflect both compensatory mutations and dependencies among epitopes. On the whole, the phylogenetic dependency network predicts that a major proportion of p17 (41%) and p24 (20%) codons are under selective pressure from at least one HLA allele, a result that confirms a dominant role of T-cell responses in driving viral evolution [3],[5],[83].

This study also represents a significant step forward by providing a statistical approach that can help differentiate direct (*H*→*A*) HLA escape polymorphisms from indirect or, more specifically, one-hop (*H*→*B*) escape polymorphisms in situations where the true interaction is the chain *H*→*A*→*B*. Although the direct–indirect distinction can arise under several mechanisms, the explicit statistical interpretation is as follows: a direct HLA-polymorphism *H*→*A* association means the HLA allele *H* is a strong predictor of the polymorphism *A*, whereas an indirect HLA-polymorphism association *H*→*A*→*B* means the polymorphism *B* is better predicted by the polymorphism *A* than by the HLA allele *H*. Although *B* is in a sense HLA-associated, the distinction of direct versus indirect associations may have important biological implications. For example, many of the indirect associations identified by the dependency network for the B*57-restricted TW10 and B*27-restricted KK10 epitopes are consistent with known compensatory mutations associated with escape in these epitopes [38],[39]. In addition to these described pathways, the dependency network reports a number of covarying amino acids. Many of these are in close physical contact, and thus likely candidates for compensatory pathways that can be tested via in vitro replication capacity assays, although distal covarying codons may also exhibit compensatory relationships [32]–[36]. Understanding the specifics of compensatory-based covariation has important implications for T-cell-based vaccine design, as escapes that require multiple compensatory mutations may take longer to arise due to chance and the compensatory mutations may not completely recover lost fitness [3],[38],[39],[41].

Compensation is not the only potential causal mechanism of codon covariation. Other mechanisms include those associated with CTL-mediated covariation. Indeed, the PDN indicates that up to 50% of the observed codon-codon covariation occurs between epitopes restricted by the same HLA allele, suggesting much of the observed codon covariation in HIV is CTL-mediated. Two possible mechanisms of CTL-mediated covariation include inter-patient variability in the immune system's ability to target epitopes and consistent patterns of epitope targeting due to immunodominance. Distinguishing between these two mechanisms may have direct relevance to vaccine design, but will require comparing the results of the PDN to clinical response data that can measure epitope targeting and longitudinal samples that can identify order of escape. Although it is well known that the order in which the epitopes of some HLA alleles are targeted is broadly consistent [60],[80],[84], identifying new patterns may yield new vaccine candidates. Specifically, it is possible that HLA alleles that are currently considered non-protective target ineffective dominant epitopes during acute infection. Redefining the immunodominance hierarchy via immunogen exposure may thus increase the effectiveness of these alleles upon subsequent HIV challenge [85].

A major challenge to vaccine design is global HIV diversity [15],[16]. Although there is accumulating evidence that suggests that patterns of escape appear to be broadly predictable [5], [6], [8]–[10],[86], these studies have been limited to relatively small sample sizes or cohorts consisting predominantly of a single clade. Although a comparison of the Durban and British Columbia results showed instances of both consistency and divergence of associated escape in the two clades [10], these studies were run separately, did not account for codon covariation, and used different methods for determining associations. Thus, the extent to which escape pathways are shared across clades was largely unknown. Our results, which reflect data equally distributed between clade B and clade C sequences and are evaluated by taking HLA LD, viral lineage and codon covariation into account, confirm the existence of common escape pathways. This similarity suggests that a broadly reactive vaccine may be possible, though more work to further characterize inter-clade similarities and differences will be necessary.

Despite the broad similarities seen between clades B and C, we noted several intriguing examples where the resistant form of an epitope matched the consensus sequence for one of the clades. Such examples support the HLA footprinting hypothesis [6],[59], which proposes that consensus sequences of circulating strains in a population are a result of consistent escape (and lack of reversion) from the most common HLA alleles in that population, an hypothesis that is especially well founded in cases where the consensus polymorphisms are different in different populations. For example, 53% of individuals in the British Columbia cohort [9] have A*01, whereas only 24% of the Durban cohort [10],[44] have A*01, and F79 (clade B consensus) is the resistant form of the association. Furthermore, alleles A*29 and A*68 have higher frequencies in the South African cohort, and Y79 (clade C consensus) is the resistant form of their associations. Thus, at codon 79, there appears to be broad selection pressure for evolutionary fixation of F in the South African cohort and fixation of Y in the British Columbian cohort. Our analyses identified a total of 21 codons (four with independent experimental support [51]) where the predicted escape matched clade B or C consensus, adding support to the hypothesis that CTL pressure serves a broad, population-level role in shaping HIV evolution, and may even serve a key role in clade differentiation [6].

We have focused this study on the highly immunogenic Gag p17 and p24 proteins, which are believed to serve a key role in effective control of HIV [44], [87]–[89]. Moving forward, it will be important to extend such studies to full length genomes, where patterns of covariation may reflect cites of protein-protein interaction [22],[90],[91] and may further reveal broad patterns of immunodominance. Furthermore, as the number and diversity of large cohorts of HIV-infected, HLA-typed, individuals continue to grow [6],[9],[10],[44],[86], it will be important to combine datasets in order to increase statistical power and further detail the similarities and differences among clades that may inform broad-coverage immunogen design.

One limitation of our two-clade study is that, because the HLA data in the HOMER cohort had only 2-digit resolution, we truncated the HLA data in the Durban cohort to 2 digit types as well. Although closely related HLA alleles often target the same or similar epitopes [51], making 2-digit resolution an appropriate choice for some allele-epitope pairs, important differences do exist. An example is the distinction between the B*5801 allele that is associated with effective viral control, and B*5802 which is associated with poor viral control [73],[92]. In cases where the prevalence of four-digit resolution types differs substantially between cohorts (as is the case with B*58) and the four-digit types target different epitopes, truncation to two-digit types before combining cohorts will lead to confounding in which the two-digit types from one cohort will tend to lead to escape whereas the two-digit types from another cohort do not. In principle, a better approach would be to include both 2-digit and 4-digit HLA alleles (or any other grouping of alleles) as predictor attributes in our model. For example, if all B*57 alleles select for the same escape mutation, then B*57 would be chosen by the model as a stronger predictor than B*5701, whereas escape mutations selected only by B*5701 would lead to the 4-digit allele being chosen. Of course, these facts should encourage researchers to perform high resolution typing on individuals in their cohorts. In addition, Listgarten et al. [93] have developed a statistical approach for inferring high resolution HLA alleles from low resolution haplotypes. Although incorporating the uncertainly of those predictions into the PDN is beyond the scope of this paper, the ability to infer high resolution HLA data will allow for more effective evaluation of large, multi-cohort studies.

The comparative method has long been used to generate hypotheses regarding traits and the environment [12]–[14]. Because (quasi-) species share a common history, the inherent population structure (in this case, the phylogeny) must be accounted for [18], and numerous methods that do so have been proposed (e.g., [12],[13],[94] and references therein). Our study on HLA immune escape mutations suggests, however, that simply accounting for population structure is not enough, as HLA linkage disequilibrium (structure among environmental predictors) and codon covariation (structure among target traits) are at least as important as phylogeny in both increasing statistical power and avoiding false positives.

This issue is relevant to applications beyond those studied here. Specifically, whenever chains of interactions are common, pairwise methods will tend to identify direct as well as indirect correlations. This effect was most dramatically seen in the synthetic codon-covariation tests, in which using a logistic regression-like approach (which accounts for chains) dramatically outperformed the phylogenetic pairwise approach. Although many phylogeny-aware comparative methods have been developed for codon-covariation [22], the problem of chains of interactions has only recently been addressed [37],[95]. The PDN provides an efficient framework in which chains of interactions can be identified in the context of both the phylogeny and confounding from external sources of selection pressure (here, HLA-mediated CTL response).

The first approach to identifying chains of interactions in a phylogenetic context was recently provided by Poon et al. [37]. They employed a *directed acyclic graphical* (DAG) model rather than a dependency network. In a DAG model, arcs from predictor to target attributes form a directed acyclic graph and local distributions take the same form as in a PDN. (A DAG model is often referred to as a Bayesian network, although the latter name is misleading as non-Bayesian procedures can be used to construct DAG models.) When learning the distributions in the DAG model, Poon et al. took phylogeny into account, although in a way different from our approach. In particular, when learning the distribution of an attribute given its parents in the DAG model, they imputed for each individual the value of the attribute corresponding to the ancestor of that individual in the phylogeny. These imputed values were then treated as observed data and fed to a standard DAG model structure learning algorithm.

The PDN provides an alternative approach that leverages the strengths of dependency networks. The most apparent difference is that dependency networks allow cycles, resulting in a network that is easier for the non-expert to interpret than is the DAG model [43]. In addition, Poon et al. used unrestricted local distributions in contrast to our use of Noisy Add. The use of Noisy Add, where the number of parameters is linear in the number of parents rather than exponential, results in a substantial increase in power. Finally, because the PDN is concerned only with local probabilities, only the target variable is conditioned on the phylogeny, allowing the PDN to efficiently model associations with attributes, such as HLA alleles, that are not expected to follow the phylogeny, as well as attributes, such as other codons, that are expected to follow the same phylogeny [21]. The result is an efficient method that can simultaneously incorporate a diverse range of selection pressure attributes.

One drawback of a dependency network relative to a DAG model is that the local distributions among the target attributes overlap and yet are learned independently. (For example, the local distribution for *A* given *B* and the local distribution for *B* given *A* are closely related, yet are learned independently.) This independent learning leads to a decrease in statistical efficiency. In practice, however, this decrease is typically minimal [43]. Another drawback of a dependency network is that the presence of cycles make inference of the joint distribution cumbersome, requiring an inefficient modified Gibbs sampling procedure to estimate the joint likelihood [43]. One possible solution is to modify the method for constructing a PDN to yield a DAG model. In particular, we can choose a random ordering for the attributes, and then build a PDN wherein the allowed predictors of a target attribute are only those that precede the target attribute in the ordering. The resulting collection of local probability distributions defines a DAG model (where acyclicity is guaranteed by the ordering constraint). The resulting model can be improved substantially by applying the above procedure to a dozen or so random orderings, and then choosing the best model according to some criterion (e.g., a Bayesian criterion or cross validation) [96]. The resulting DAG is a generative model that can be used to perform inference on the joint distribution.

The purpose of conditioning each target attribute on phylogeny is to account for population structure that renders some sequences naturally more similar to each other. In the case of comparative genomics across (quasi-) species, a phylogeny is a reasonable model of the population structure. Even among individuals of the same species, a phylogeny may be a good representation of the structure, insofar as the structure reflects a hierarchical clustering of the individuals that may be due to a number of genetic and environmental factors [21],[97],[98]. It may prove useful, however, to extend the spirit of the PDN to non-phylogenetic models of population structure. For example, in genome wide association studies (GWAS), a number of recent approaches have been described that use models of population structure that are richer than a hierarchical cluster [99]–[105]. Most of these methods focus on pairwise comparisons, though methods for more complex interactions also exist (see e.g. [106]–[108]). Dependency networks that use appropriate models of background structure coupled with Noisy Add and forward selection may allow simultaneous correction for population structure and interacting complex traits.

### Conclusion

We have introduced phylogenetic dependency networks for modeling multiple sources of selection pressure on evolutionary traits, and have applied this approach to the characterization of patterns of immune escape in HIV. In so doing, we have identified broad patterns of covariation and CTL adaptation, the verification of which should broadly inform vaccine design. Although the specific distributions described here may not be suited for all applications, dependency networks that are conditioned on appropriate models of population structure are widely applicable, and represent a powerful tool for efficiently combining multiple effects in population-based analyses. The programs used in this paper are available at http://www.codeplex.com/MSCompBio.

## Supporting Information

Table S1 List of associations with *q*<0.2.
(0.29 MB XLS)

Table S2 Distance in Angstroms between codons with *q*<0.2.
(0.08 MB XLS)
